# Supplementary material for: Epoxy-oxylipins direct monocyte fate in inflammatory resolution in humans
Source: Nat Commun. 2026 Jan 16;17:431. doi: 10.1038/s41467-025-67961-5 (PMC12811254; doi:10.1038/s41467-025-67961-5)
Supplement: Supplementary file 1 — Supplementary Information [file 41467_2025_67961_MOESM1_ESM.pdf]

Supplementary Materials

- Supplementary Table 1 – Cytokines and chemokine levels in blister fluid with prophylactic and therapeutic sEH inhibition.
- Supplementary Table 2 - Table of antibodies used for Flow Cytometry
- Supplementary Table 3 - Table of antibodies used for immunohistochemistry
- Supplemental Table 4 - Details of oxylipin analysis by LCMS.
- Supplementary figures

A Blister fluid (prophylactic)

Cytokine	4 hr Blister Untreated	4 hr Blister Treated	24 hr Blister Untreated	24 hr Blister Treated
TNFa	5770.61 +/- 6043.72	6623.62 +/- 2687.49	1753.68 +/- 3606.08	249.60 +/- 97.58
IL-10	4462.07 +/- 1619.61	4614.98 +/- 2970.57	2476.65 +/- 1252.78	2686.17 +/- 1255.25
IL-1B	929.51 +/- 632.77	983.84 +/- 512.10	828.24 +/- 1666.27	98.08 +/- 50.02
IL-8	4850.06 +/- 1935.69	3697.13 +/- 2285.54	1916.58 +/- 4296.83	172.83 +/- 104.44
CCL2	9784.31 +/- 2964.26	11325.21 +/- 5783.37	5479.48 +/- 3539.92	3630.42 +/- 1350.96
CX3CL1	2415.38 +/- 568.15	2951.71 +/- 919.69	1641.72 +/- 618.15	1525.01 +/- 289.61
CXCL10	6910.95 +/- 493.70	8328.63 +/- 4285.09	6783.12 +/- 464.07	6725.70 +/- 372.77
IL-17E	85.73 +/- 98.18	169.82 +/- 195.92	72.36 +/- 59.37	176.82 +/- 121.40
VEGF	944.98 +/- 513.37	1757.22 +/- 632.37	334.53 +/- 625.51	102.36 +/- 29.20
CCL5	1605.39 +/- 835.08	2465.39 +/- 603.47		

A Blister fluid (therapeutic)

Cytokine	24 hr Blister Untreated	24 hr Blister Treated	48 hr Blister Untreated	48 hr Blister Treated
TNFa	302.66 +/- 105.54	363.11 +/- 89.65	134.66 +/- 66.06	157.85 +/- 106.79
IL-10	2861.99 +/- 509.05	2938.51 +/- 773.86	1922.51 +/- 682.28	2394.15 +/- 942.87
IL-1B	172.56 +/- 103.91	153.91 +/- 71.24	33.56 +/- 27.72	46.45 +/- 47.96
IL-8	212.55 +/- 77.75	341.49 +/- 215.95	189.27 +/- 118.30	1554.25 +/- 4427.92
CCL2	3646.41 +/- 1561.72	4321.93 +/- 1418.11	2432.15 +/- 933.10	2593.43 +/- 812.96
CX3CL1	1855.90 +/- 391.97	2103.13 +/- 385.96	954.59 +/- 408.35	1212.09 +/- 695.53
CXCL10	8526.37 +/- 348.10	8726.21 +/- 740.57	9579.92 +/- 3230.12	10514.04 +/- 3195.06
IL-17E	315.89 +/- 244.45	152.18 +/- 137.59	446.14 +/- 210.68	531.29 +/- 438.59
VEGF	185.33 +/- 93.64	186.44 +/- 129.34	91.78 +/- 43.05	127.53 +/- 103.52

Supplementary Table 1 – Cytokines and chemokine levels in blister fluid with prophylactic and therapeutic sEH inhibition.

Fluorophore	Marker	Category Code	Concentration	Target
**FITC**	CD3	300306	1:100	T cell
**FITC**	CD19	302206	1:100	B cell
**FITC**	CD20	302304	1:100	B cell
**FITC**	CD56	392414	1:100	NK cell
**FITC**	CD66b	305104	1:100	Neutrophil
**PerCP-Cy5.5**	CD56	318322	1:100	NK cell
**PerCP-Cy5.5**	CD141	344112	1:100	DC
**PerCP-Cy5.5**	CD11b	301328	1:100	
**BV421**	CD11c	301628	2.5:100	Mononuclear Phagocyte
**BV421**	CD62L	304828	1:50	
**BV510**	CCR2	357218	2.5:100	Monocyte
**BV510**	CD11b	301628	1:100	Mononuclear phagocyte
**BV510**	HLA-DR	307646	1:100	Mononuclear phagocyte
**BV605**	CD86	374214	1:100	Monocyte/DC
**BV605**	CD25	302632	1:50	T cell
**BV711**	CD16	302044	1:100	Monocyte/ Granulocyte
**BV785**	CD206	321142	1:50	Monocyte/Macrophage
**BV785**	CD163	333632	1:100	Mononuclear phagocyte
**BV786**	CD27	563327	1:100	T cell
**BUV395**	CD123	564195	1:100	pDC
**BUV395**	CD4	564724	1:100	T cell
**BUV395**	CD1a	756536	1:100	DC/Langerhan
**APC**	CD163	333610	3:100	Monocyte/Macrophage
**APC**	CD207	352205	1:100	Langerhan
**AF700**	HLA-DR	307626	2.5:100	Mononuclear Phagocyte
**AF700**	CD66b	305114	1:50	Neutrophil
**APC-Cy7**	CD1c	331520	2.5:100	DC
**APC-Cy7**	CD8	301016	1:100	T cell
**APC-Cy7**	CD206	321120	1:100	Mononuclear phagocyte
**PE**	CD205	359204	2.5:100	Monocyte/DC
**PE**	FOXP3	320107	1:10	T cell
**PE**	MerTK	367608	1:20	Mononuclear Phagocyte
**PE**	CCR7	535204	1:100	
**PE**	CD25	302605	1:100	T cell
**PE-Dazzle**	CD19	302252	1:100	B cell
**PE-Dazzle**	CD20	302348	1:100	B cell
**PE-Dazzle**	CD3	300336	1:100	T cell
**PE-Dazzle**	CD56	392410	1:100	NK cell
**PE-Dazzle**	CD66b	305122	1:100	Neutrophil
**PE-Dazzle**	CD64	305032	1:100	Monocyte
**PE-Cy7**	TIM-4	354006	1:100	Monocyte/Macrophage
**PE-Cy7**	CD62L	304822	1:100	
**PE-Cy7**	CD45RA	304125	1:100	T cell
**BUV805**	CD14	612902	1:100	Monocyte
**BUV805**	CD69	748763	1:100	Activation
**Zombie UV**	Live/Dead	423107	1:1000	
**AF647**	P-p38	526066	1:10	

Supplementary Table 2 - Table of antibodies used for Flow Cytometry

Fluorophore	Marker	Category Code	Concentration	Target
**Starbright UV 665**	CD45RO	MCA461SBUV665	1:50	T cell
**BUV615**	HLA-DR	751142	1:100	
**BUV737**	CD103	748502	1:50	T cell
**BV421**	CCR6	562515	1:20	
**BV480**	CD45RA	566155	1:100	T cell
**BV650**	CD16	563692	1:100	Monocyte
**BV711**	CTLA4/CD152	369632	1:50	
**FITC**	CLA	321306	1:100	
**PercPFluor710**	TIGIT	46-9500-42	1:100	
**RB780**	CD39	570123	1:100	
**AF700**	CD14	325614	1:100	Monocyte
**APC-H7**	CD3	560275	1:100	T cell
**PE**	FoxP3	12-4777-43	1:100	T reg cell
**PE-Dazzle**	Ki67	350533	1:100	
**Zombie NIR**	Live/Dead	423106	1:1000	

Supplementary Table 2 - Table of antibodies used for Flow Cytometry cont.

Primary Antibody	Clone	Host	Reactivity	Dilution	Supplier
**CYP2C8,9,18, 19**	Polyclonal	Rabbit	Human	1:150	Biorbyt
**CYP2J2**	Polyclonal	Rabbit	Human	1:300	Abcam
**mEH**	Polyclonal	Rabbit	Human	1:200	Biorbyt
**sEH**	Polyclonal	Rabbit	Human	1:150	Biorbyt
**EH3**	Polyclonal	Rabbit	Human	1:200	Biorbyt

Supplementary Table 3 - Table of antibodies used for immunohistochemistry

Analyte	Precursor (m/z)	Product (m/z)	RT (min)	ISTD	Low Standard (pg/ul)	High Standard (pg/ul)	r^2
11,12-DHET	337.2	167.2	7.23	11,12-DHET-d11	0.1	120	0.995
11,12-DiHETE	335.2	167.1	5.99	11,12-DHET-d11	0.1	120	0.99
11,12-EET	319.2	179.2	11.99	11,12-EET-d11	0.2	240	0.996
11-HETE	319.2	167.2	10.04	15-HETE-d8	0.1	120	0.994
12,13-DHOME	313.2	183.2	6.08	11,12-DHET-d11	1	1200	0.985
12,13-EpOME	295.2	195.2	11.2	11,12-EET-d11	0.5	600	0.992
12-HETE	319.2	179.2	10.32	15-HETE-d8	1.25	1500	0.995
13,14-DiHDPA	361.2	193.1	7.36	11,12-DHET-d11	0.1	120	0.994
13-HODE	295.2	195.2	9.04	15-HETE-d8	2.5	3000	0.993
14,15-DHET	337.2	207.2	6.69	11,12-DHET-d11	0.1	120	0.992
14,15-DiHETE	335.2	207.1	5.8	11,12-DHET-d11	0.1	120	0.993
14,15-EET	319.2	219.3	11.39	11,12-EET-d11	0.2	240	0.994
15-HETE	319.2	219.1	9.62	15-HETE-d8	0.1	120	0.994
16,17-DiHDPA	361.2	233.1	7.12	11,12-DHET-d11	0.1	120	0.994
16,17-EpDPA	343.2	233.1	11.77	11,12-EET-d11	0.2	240	0.995
17,18-DiHETE	335.2	247.1	5.57	11,12-DHET-d11	0.1	120	0.991
17,18-EpETE	317.2	259.1	9.65	11,12-EET-d11	0.1	120	0.995
17-HDHA	343.2	281.2	9.91	15-HETE-d8	0.025	30	0.992
19,20-DiHDPA	361.2	273.2	6.76	11,12-DHET-d11	0.1	120	0.994
19,20-EpDPE	343.2	281.1	11.35	11,12-EET-d11	0.2	240	0.994
19-HETE	319.2	231.1	8.06	15-HETE-d8	0.5	600	0.993
20-HETE	319.2	245.1	8.23	15-HETE-d8	0.5	600	0.991
22-HDoHE	343.2	269.1	8.99	15-HETE-d8	0.025	30	0.994
5,6-DHET	337.2	145.1	12.3	11,12-DHET-d11	0.1	120	0.994
5-HETE	319.2	257.3	10.86	15-HETE-d8	0.1	120	0.993
8,9-DHET	337.2	127.3	7.66	11,12-DHET-d11	0.1	120	0.994
8,9-EET	319.2	151.1	12.2	11,12-EET-d11	0.2	240	0.993
9,10-DHOME	313.2	201.1	6.35	11,12-DHET-d11	1	1200	0.972
9,10-EpOME	295.2	277.3	11.42	11,12-EET-d11	0.5	600	0.998
9-HODE	295.2	171.2	9.19	15-HETE-d8	2.5	3000	0.994

Supplementary Table 4 - Details of oxylipin analysis by LCMS.

Ionization masses (Precursor and Product) in mass/charge ratio (m/z), retention time (RT), internal standard (ISTD) used, and the minimum and maximum concentrations used in the standard curve (pg/µl injected) and r2 of the linear standard curves is indicated. Analyte quantification was determined using standard curves determined based on peak area analyte/peak area of indicated ISTDs. The lower limit of quantification LLOQ was at or above the lowest standard used for each curve (S/N>10).

SUPPLEMENTARY FIGURES

Supplementary, Figure 1

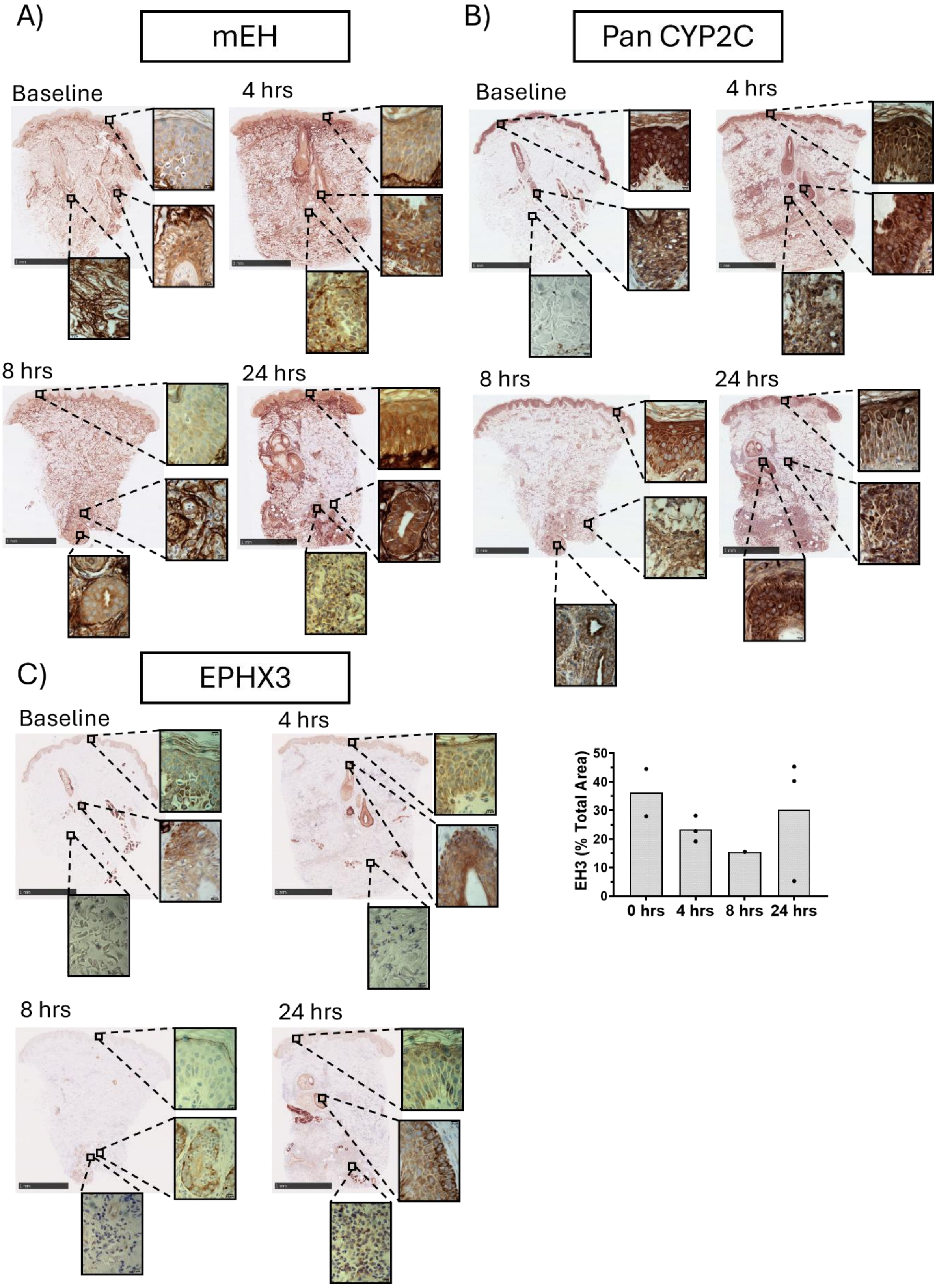

Supplementary Figure 1 – Expression of CYP2C, mEH and EH3 during inflammation in human tissue

Paraffin-embedded skin punch biopsies were obtained at baseline, 4, 8 and 24 hrs post UVKEc injection.

Sections were incubated with (A) mEH, (B) Pan CYP2C8/9/18/19 and (C) EPHX3 primary antibodies and visualized by avidin-biotin complex-based detection methods. The sections were subsequently counterstained with haematoxylin.

(C) Skin biopsies at baseline, 4, 8 and 24 hrs were stained and the % area of staining quantified for EPHX3 (n =1-3; biologically independent samples).

Supplementary, Figure 2

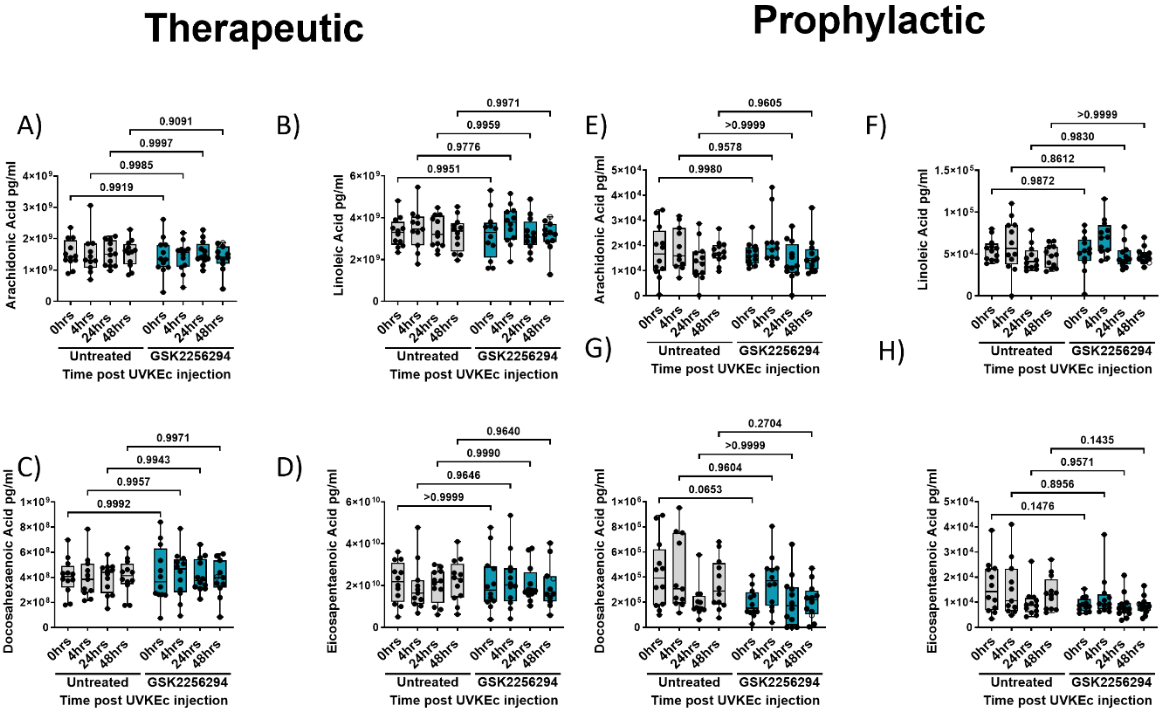

Supplementary Figure 2 – Levels of PUFAs in peripheral blood with prophylactic and therapeutic dosing of GSK2256294 during inflammation.

The forearms of participants were intradermally injected with UV-killed *E. coli* (UVKEc) resulting in a local and peripheral inflammatory response. 2 hrs prior to (prophylactic) or 4 hrs after (therapeutic) UVKEc injection, participants were dosed with 15 mg of GSK2256294. Plasma was collected and subject to lipidomic analysis at all timepoints. Levels of (A) Arachidonic Acid (AA), (B) Linoleic Acid, (C) Docosahexaenoic Acid (DHA), and (D) Eicosapentaenoic Acid (EPA) in the therapeutic arm of the study (untreated: n = 11-12; GSK2256294: n = 11-12; biologically independent samples).

Levels of (E) Arachidonic Acid (AA), (F) Linoleic Acid, (G) Docosahexaenoic Acid (DHA), and (H) Eicosapentaenoic Acid (EPA) in the prophylactic arm of the study (untreated: n = 11-12; GSK2256294: n = 12; biologically independent samples). All data are expressed in pg/ml. Box-and-whisker plots show the median (center line), the interquartile range (25th–75th percentiles; box), and the full data range (whiskers, minimum to maximum). Normality was assessed using the D’Agostino & Pearson test, Shapiro-Wilk test and visualised using a QQ plot. Data were analysed using two-way-ANOVA mixed effect analysis with Šídák’s multiple comparison test. Source data are provided as a Source Data file.

Supplementary, Figure 3

Prophylactic

Lipid	0hr Untreated	4hr Untreated	24hr Untreated	48hr Untreated	0hr Treated	4hr Treated	24hr Treated	48hr Treated
11,12-DHET	136.67 +/- 50.45	118.83 +/- 50.70	114.98 +/- 32.86	132.27 +/- 38.77	131.65 +/- 72.56	150.79 +/- 46.09	114.75 +/- 31.40	114.73 +/- 35.40
11,12-EET	20.85 +/- 10.86	20.65 +/- 12.95	13.77 +/- 6.03	17.94 +/- 7.64	15.94 +/- 5.55	27.46 +/- 12.95	17.02 +/- 5.99	17.85 +/- 7.20
11-HETE	221.90 +/- 148.48	123.63 +/- 56.16	277.23 +/- 117.22	449.48 +/- 229.83	136.52 +/- 32.71	153.15 +/- 55.66	256.58 +/- 112.66	384.10 +/- 218.91
12,13-DHOME	2901.48 +/- 1284.35	4283.38 +/- 3067.55	2290.17 +/- 1096.67	2376.21 +/- 2033.26	1465.33 +/- 1043.09	1916.15 +/- 3085.74	1173.13 +/- 1158.00	1530.13 +/- 2051.44
12,13-EpOME	1297.31 +/- 1065.40	2225.63 +/- 1586.25	1635.79 +/- 2652.96	1243.02 +/- 1588.07	5259.10 +/- 6758.25	6289.48 +/- 2777.48	3161.10 +/- 2646.04	3386.48 +/- 1560.72
12-HETE	2174.42 +/- 2444.80	1041.44 +/- 1419.34	1232.44 +/- 980.04	1567.75 +/- 776.62	1534.08 +/- 1562.50	1444.25 +/- 1407.83	1690.23 +/- 996.33	1346.60 +/- 779.58
13,14-DiHDPA	52.88 +/- 28.24	49.25 +/- 27.53	43.10 +/- 13.85	49.58 +/- 23.17	36.69 +/- 11.05	43.21 +/- 24.95	34.58 +/- 13.95	34.92 +/- 23.04
13-HODE	9396.33 +/- 11192.94	7344.69 +/- 7111.88	14138.52 +/- 13143.27	13564.10 +/- 11342.64	4522.35 +/- 4511.11	5071.58 +/- 7637.86	8499.75 +/- 13463.04	12321.46 +/- 11063.73
14,15-DHET	157.90 +/- 60.79	60.63 +/- 66.96	122.38 +/- 30.42	141.27 +/- 38.17	102.94 +/- 50.92	84.83 +/- 67.34	110.83 +/- 45.10	100.40 +/- 38.57
14,15-DiHETE	88.38 +/- 33.06	87.56 +/- 49.18	73.19 +/- 17.78	74.75 +/- 26.10	61.67 +/- 14.83	75.67 +/- 49.26	59.65 +/- 17.40	50.75 +/- 27.84
14,15-EET	32.63 +/- 18.34	29.00 +/- 16.26	23.90 +/- 6.07	29.98 +/- 11.17	41.69 +/- 22.41	61.77 +/- 17.44	42.40 +/- 6.81	43.00 +/- 13.69
15-HETE	215.17 +/- 92.09	164.06 +/- 65.67	232.67 +/- 90.45	360.85 +/- 158.20	183.19 +/- 46.23	199.33 +/- 65.96	250.15 +/- 86.55	327.94 +/- 141.83
16,17-DiHDPA	54.08 +/- 34.37	48.75 +/- 27.71	40.40 +/- 12.07	46.48 +/- 21.59	35.35 +/- 11.07	39.75 +/- 23.37	33.17 +/- 11.61	31.79 +/- 21.90
17,18-DiHETE	1284.46 +/- 469.70	1311.35 +/- 858.31	1054.88 +/- 298.91	1023.98 +/- 391.05	856.90 +/- 226.68	1012.21 +/- 858.12	829.58 +/- 317.74	712.40 +/- 360.78
17-HDHA	776.08 +/- 467.80	478.90 +/- 193.58	900.96 +/- 357.89	1703.56 +/- 1090.71	414.67 +/- 199.04	449.50 +/- 195.83	652.48 +/- 380.05	949.90 +/- 1107.07
18-HEPE	309.79 +/- 160.43	284.71 +/- 128.89	356.73 +/- 135.71	470.88 +/- 273.44	206.98 +/- 57.38	231.79 +/- 136.03	270.21 +/- 132.29	325.27 +/- 270.35
19,20-DiHDPA	767.96 +/- 495.38	713.92 +/- 475.09	642.38 +/- 279.46	679.48 +/- 297.72	466.15 +/- 167.04	601.08 +/- 385.97	520.15 +/- 284.72	458.56 +/- 298.37
19,20-EpDPE	526.06 +/- 334.74	269.31 +/- 129.49	651.31 +/- 311.70	1213.85 +/- 686.70	227.75 +/- 93.93	301.69 +/- 128.67	429.85 +/- 320.92	690.23 +/- 685.59
19-HETE	87.96 +/- 46.14	67.08 +/- 36.35	63.90 +/- 26.54	75.46 +/- 23.36	70.13 +/- 17.79	90.13 +/- 30.59	66.85 +/- 27.44	64.94 +/- 23.31
20-carboxy-AA	227.83 +/- 91.78	134.77 +/- 62.08	332.10 +/- 93.93	424.52 +/- 141.23	222.02 +/- 126.93	207.48 +/- 68.46	347.63 +/- 81.83	436.17 +/- 130.85
20-HETE	202.08 +/- 146.06	181.27 +/- 163.93	153.75 +/- 75.21	175.88 +/- 102.57	115.88 +/- 25.82	171.98 +/- 161.80	113.17 +/- 75.41	130.67 +/- 100.96
22-HDoHE	272.56 +/- 206.82	233.44 +/- 201.91	171.23 +/- 75.34	229.23 +/- 135.97	159.19 +/- 62.35	214.81 +/- 187.11	138.60 +/- 75.36	152.13 +/- 132.19
5,6-DHET	106.56 +/- 29.78	95.44 +/- 34.25	89.96 +/- 23.66	100.67 +/- 26.44	99.90 +/- 20.10	113.25 +/- 34.17	99.56 +/- 24.83	114.00 +/- 28.86
5-HETE	388.33 +/- 207.22	264.29 +/- 111.96	458.21 +/- 179.47	680.04 +/- 280.40	252.75 +/- 61.18	304.54 +/- 112.03	410.29 +/- 171.78	622.15 +/- 254.77
8,9-DHET	58.54 +/- 19.93	54.35 +/- 21.63	49.75 +/- 14.75	48.44 +/- 12.60	46.38 +/- 17.14	59.08 +/- 19.64	48.13 +/- 15.13	48.08 +/- 11.62
8-HETE	2485.00 +/- 1293.98	1511.13 +/- 646.25	3305.71 +/- 1277.48	5074.42 +/- 2287.69	1691.13 +/- 498.98	1969.79 +/- 644.57	3182.60 +/- 1236.72	4552.27 +/- 2141.61
9,10-DHOME	2544.92 +/- 2271.53	7103.46 +/- 11540.68	1781.40 +/- 1308.87	1601.92 +/- 1527.04	1288.10 +/- 1352.51	2263.46 +/- 11554.31	944.31 +/- 1346.41	1130.63 +/- 1532.52
9,10-EpOME	498.31 +/- 636.28	1105.19 +/- 1558.48	467.96 +/- 610.74	417.19 +/- 363.04	686.63 +/- 1163.24	1022.56 +/- 1629.76	554.65 +/- 606.36	603.50 +/- 352.33
9-HODE	11929.02 +/- 9291.57	20016.40 +/- 19000.39	13426.79 +/- 10465.73	15972.58 +/- 8331.88	11000.88 +/- 9249.59	13110.92 +/- 18891.89	9258.58 +/- 10401.76	13408.52 +/- 7802.38

Therapeutic

Lipid	0hr Untreated	4hr Untreated	24hr Untreated	48hr Untreated	0hr Treated	4hr Treated	24hr Treated	48hr Treated
11,12-DHET	297.92 +/- 66.43	289.70 +/- 95.21	263.69 +/- 51.31	267.52 +/- 67.83	275.77 +/- 86.13	362.16 +/- 249.03	231.72 +/- 55.87	240.86 +/- 67.05
11,12-EET	54.63 +/- 5.54	56.40 +/- 10.41	51.96 +/- 8.55	53.95 +/- 12.32	52.47 +/- 6.54	58.80 +/- 24.27	55.40 +/- 13.29	59.88 +/- 10.88
11-HETE	298.38 +/- 103.63	313.87 +/- 182.64	269.60 +/- 95.82	285.03 +/- 111.59	304.93 +/- 123.62	331.51 +/- 179.49	255.87 +/- 101.77	258.40 +/- 87.48
12,13-DHOME	7199.11 +/- 8750.16	5674.35 +/- 2871.51	9405.65 +/- 10893.17	8047.75 +/- 8005.57	2697.58 +/- 2019.86	7905.87 +/- 4753.78	1900.64 +/- 1449.39	2489.24 +/- 2061.88
12,13-EpOME	3498.30 +/- 4288.44	4094.06 +/- 2999.58	4496.60 +/- 5290.38	5055.14 +/- 9416.43	1626.15 +/- 1609.07	4210.08 +/- 2448.30	6807.48 +/- 7261.40	12471.06 +/- 16713.12
12-HETE	2115.60 +/- 761.97	2399.77 +/- 1774.33	2746.39 +/- 2018.25	2179.21 +/- 1538.53	2139.50 +/- 1569.73	6627.51 +/- 11563.62	2178.49 +/- 1154.09	1789.47 +/- 1070.10
13,14-DiHDPA	113.05 +/- 31.39	105.06 +/- 29.25	96.00 +/- 29.37	95.07 +/- 30.94	120.54 +/- 36.84	143.54 +/- 67.78	104.63 +/- 32.64	108.49 +/- 40.54
13-HODE	71167.01 +/- 93504.61	64382.37 +/- 53850.63	85876.06 +/- 113347.24	77908.46 +/- 114331.03	20331.29 +/- 14636.91	55645.92 +/- 37910.68	26619.01 +/- 32674.45	36418.74 +/- 40385.22
14,15-DHET	720.54 +/- 126.94	736.60 +/- 180.07	647.96 +/- 118.49	643.07 +/- 140.10	730.19 +/- 193.78	847.96 +/- 485.69	621.22 +/- 149.76	626.07 +/- 181.95
14,15-DiHETE	484.31 +/- 195.02	487.94 +/- 162.16	424.40 +/- 172.90	430.10 +/- 154.98	525.04 +/- 197.75	552.76 +/- 208.32	518.58 +/- 139.70	486.87 +/- 192.32
14,15-EET	135.46 +/- 24.65	137.80 +/- 33.50	132.31 +/- 22.39	136.73 +/- 18.81	142.01 +/- 47.14	159.14 +/- 71.36	189.80 +/- 30.27	194.48 +/- 33.61
15-HETE	630.36 +/- 201.86	608.58 +/- 224.90	560.42 +/- 112.20	582.06 +/- 177.47	673.98 +/- 205.09	729.46 +/- 265.38	582.40 +/- 177.82	557.70 +/- 200.69
16,17-DiHDPA	214.50 +/- 86.15	186.22 +/- 98.47	205.65 +/- 57.06	197.58 +/- 49.73	265.22 +/- 80.64	310.76 +/- 179.81	251.74 +/- 95.05	212.24 +/- 100.63
16,17-EpDPA	8.76 +/- 6.59	6.77 +/- 5.92	6.42 +/- 4.49	7.53 +/- 5.81	6.76 +/- 4.48	12.16 +/- 10.26	10.12 +/- 4.70	10.62 +/- 6.26
17,18-DiHETE	5327.85 +/- 1863.43	4890.58 +/- 1528.35	4586.01 +/- 1850.24	4670.83 +/- 1933.60	5428.40 +/- 1790.72	5968.92 +/- 2390.01	5650.09 +/- 1982.79	5579.31 +/- 2320.80
17,18-EpETE	26.66 +/- 15.79	26.90 +/- 15.27	21.73 +/- 10.58	25.51 +/- 12.01	38.95 +/- 46.74	43.45 +/- 41.39	27.17 +/- 17.81	32.93 +/- 16.13
17-HDHA	614.84 +/- 271.40	568.00 +/- 266.54	502.25 +/- 192.94	604.93 +/- 294.30	753.53 +/- 288.22	784.31 +/- 286.74	542.13 +/- 154.64	569.20 +/- 218.91
19,20-DiHDPA	1333.80 +/- 434.45	1246.47 +/- 352.98	1145.03 +/- 357.58	1111.86 +/- 343.01	1436.90 +/- 572.54	1799.11 +/- 1115.39	1453.02 +/- 546.38	1386.41 +/- 562.86
19,20-EpDPE	286.65 +/- 140.44	295.41 +/- 138.80	234.74 +/- 112.37	259.86 +/- 110.35	335.37 +/- 168.41	375.81 +/- 228.45	230.91 +/- 91.57	268.88 +/- 104.98
19-HETE	347.53 +/- 117.81	334.88 +/- 154.36	290.43 +/- 85.84	304.21 +/- 128.49	342.39 +/- 113.74	427.68 +/- 347.58	312.88 +/- 64.71	283.81 +/- 79.17
20-HETE	613.01 +/- 267.54	622.08 +/- 250.64	515.99 +/- 250.31	556.25 +/- 258.06	621.05 +/- 286.64	932.23 +/- 967.64	539.20 +/- 175.81	581.59 +/- 195.99
22-HDoHE	692.81 +/- 472.69	641.65 +/- 344.65	588.10 +/- 401.03	578.95 +/- 434.97	705.36 +/- 298.95	1006.04 +/- 747.54	554.12 +/- 175.66	621.94 +/- 221.45
5,6-DHET	194.94 +/- 65.10	183.81 +/- 58.63	161.05 +/- 45.31	165.99 +/- 42.69	217.60 +/- 64.76	260.42 +/- 161.43	227.68 +/- 64.04	236.83 +/- 91.76
5-HETE	664.41 +/- 201.19	681.22 +/- 345.93	634.91 +/- 277.15	626.83 +/- 286.91	726.02 +/- 229.62	849.57 +/- 357.25	685.16 +/- 334.29	599.58 +/- 208.14
8,9-DHET	286.27 +/- 81.70	273.01 +/- 84.98	254.34 +/- 64.99	250.00 +/- 57.53	281.59 +/- 61.67	370.24 +/- 257.67	264.29 +/- 48.77	262.20 +/- 73.30
8,9-EET	163.31 +/- 62.06	151.44 +/- 83.53	153.91 +/- 65.30	142.60 +/- 52.38	130.78 +/- 48.71	167.52 +/- 134.29	124.91 +/- 53.33	148.75 +/- 55.21
8-HETE	4491.92 +/- 1358.56	4415.49 +/- 2278.26	4089.46 +/- 1163.26	4163.35 +/- 1389.19	4321.26 +/- 1488.35	4832.95 +/- 2401.41	3750.38 +/- 965.47	3947.17 +/- 1076.53
9,10-DHOME	7702.51 +/- 10872.12	6537.89 +/- 5167.69	8667.30 +/- 11896.70	8839.50 +/- 12239.70	1829.34 +/- 1792.33	5999.53 +/- 4616.08	1811.01 +/- 2032.68	2425.65 +/- 2457.76
9,10-EpOME	1128.83 +/- 1227.68	1200.93 +/- 875.35	1302.30 +/- 1483.98	1723.68 +/- 3191.92	496.00 +/- 462.63	1002.85 +/- 561.32	1150.26 +/- 938.12	1765.72 +/- 1680.72
9-HODE	52812.76 +/- 62837.09	58807.70 +/- 40635.86	59976.53 +/- 74225.56	61541.24 +/- 103677.64	24826.79 +/- 27809.08	59733.08 +/- 42784.63	19618.96 +/- 20114.75	35214.75 +/- 39553.89

Supplementary Figure 3 - Changes to epoxy-oxylipin levels in peripheral blood with prophylactic and therapeutic dosing with GSK2256294 during inflammation.

The forearms of participants were intradermally injected with UV-killed *E. coli* (UVKEc) resulting in a local and peripheral inflammatory response. 2 hrs prior to (prophylactic) or 4 hrs after (therapeutic) UVKEc injection, participants were dosed with 15 mg of GSK2256294. Plasma was collected and subject to lipidomic analysis at all timepoints. Data are presented as mean ± SD. In the therapeutic arm of the study each data point is representative of: (untreated: n = 11-12; GSK2256294: n = 11-12; biologically independent samples). In the prophylactic arm of the study each data point is representative of: (untreated: n = 11-12; GSK2256294: n = 12; biologically independent samples).

Supplementary, Figure 4

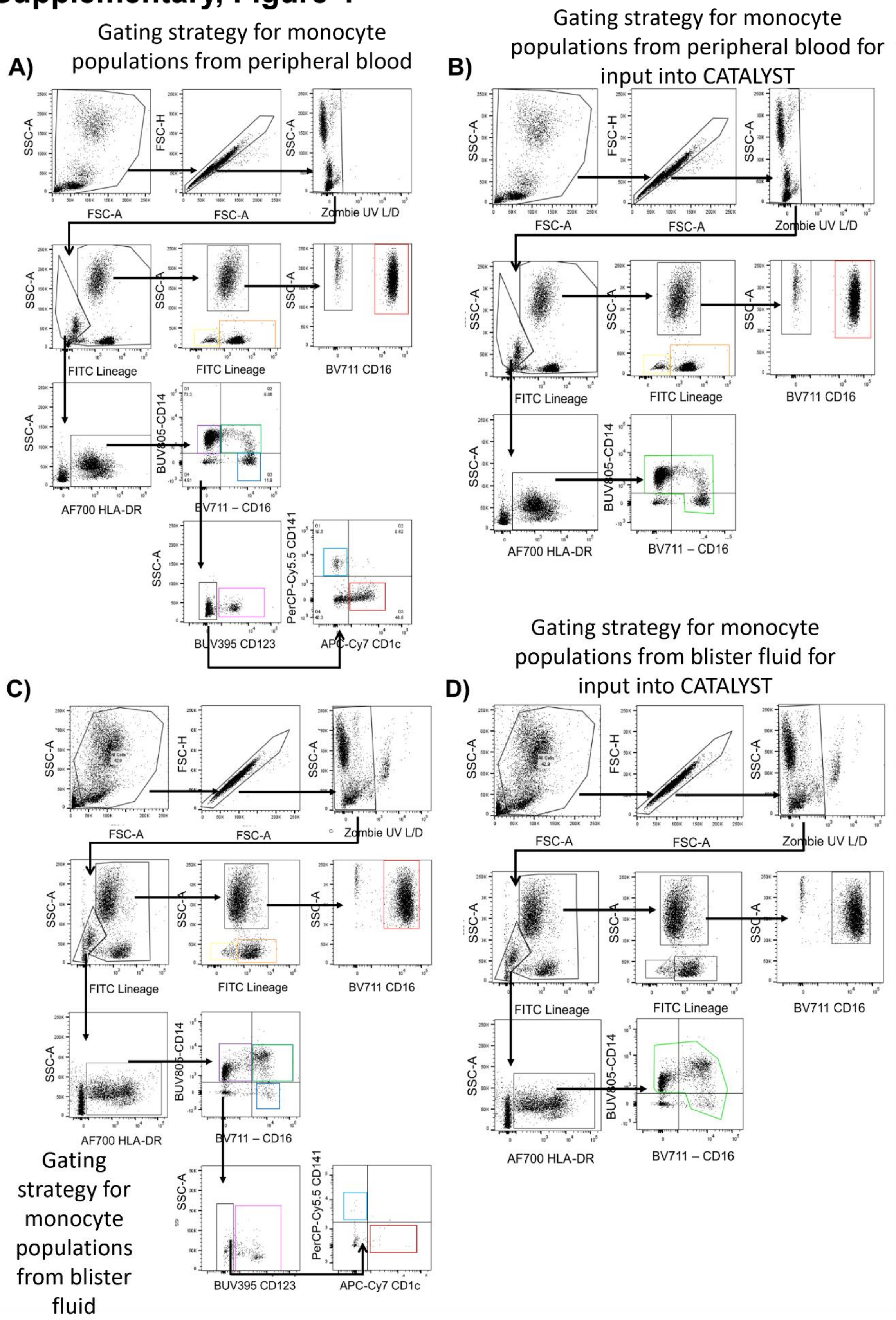

Supplementary Figure 4 - Flow cytometry gating strategies for identification of monocyte subsets in peripheral blood and blister.

(A) Identification of monocyte subsets from peripheral blood for analysis in FlowJo. An initial gate around all leukocytes assigned. These cells are taken forward to exclude doublets and dead cells. NK cells, T cells, B cells and neutrophils are removed from the sample by gating for the FITC positive cells, which includes markers for CD3, CD56, CD19, CD20 and CD66b. T cells and B cells are gated in orange and NK cells in yellow. Neutrophils are assigned based on high SSC and high CD16 expression. The FITC negative cells are taken forward and gated for AF700 HLA-DR positive cells. Classical (CD14+CD16-), Intermediate (CD14+CD16+) and Non-Classical (CD14-CD16+) subsets are assigned according to CD14 and CD16 expression. pDCs are taken from the CD14-CD16- and assigned as CD123+. cDCs are taken from the CD123- cells and assigned according to expression of CD141 (cDC1) and CD1c (cDC2).

(B) Identification of HLA-DR+CD14+CD16+ monocytes from peripheral blood for input into CATALYST for supervised clustering. An initial gate around all leukocytes assigned. These cells are taken forward to exclude doublets and dead cells. NK cells, T cells, B cells and neutrophils are removed from the sample by gating for the FITC positive cells, which includes markers for CD3, CD56, CD19, CD20 and CD66b. T cells and B cells are gated in orange and NK cells in yellow. Neutrophils are assigned based on high SSC and high CD16 expression. The FITC negative cells are taken forward and gated for AF700 HLA-DR positive cells. Monocytes that are used in the supervised clustering are gated in green.

(C) Identification of monocyte subsets from the blister for analysis in FlowJo. An initial gate around all leukocytes assigned. These cells are taken forward to exclude doublets and dead cells. NK cells, T cells, B cells and neutrophils are removed from the sample by gating for the FITC positive cells, which includes markers for CD3, CD56, CD19, CD20 and CD66b. T cells and B cells are gated in orange and NK cells in yellow. Neutrophils are assigned based on high SSC and high CD16 expression. The FITC negative cells are taken forward and gated for AF700 HLA-DR positive cells. Classical (CD14+CD16-), Intermediate (CD14+CD16+) and Non-Classical (CD14-CD16+) subsets are assigned according to CD14 and CD16 expression. pDCs are taken from the CD14-CD16- and assigned as CD123+. cDCs are taken from the CD123- cells and assigned according to expression of CD141 (cDC1) and CD1c (cDC2).

(D) Identification of HLA-DR+CD14+CD16+ monocytes from the blister for input into CATALYST for supervised clustering. An initial gate around all leukocytes assigned. These cells are taken forward to exclude doublets and dead cells. NK cells, T cells, B cells and neutrophils are removed from the sample by gating for the FITC positive cells, which includes markers for CD3, CD56, CD19, CD20 and CD66b. T cells and B cells are gated in orange and NK cells in yellow. Neutrophils are assigned based on high SSC and high CD16 expression. The FITC negative cells are taken forward and gated for AF700 HLA-DR positive cells. Monocytes that are used in the supervised clustering are gated in green.

Supplementary, Figure 5

Prophylactic

Cell Type	0hr Untreated	4hr Untreated	24hr Untreated	48hr Untreated	0hr Treated	4hr Treated	24hr Treated	48hr Treated
Neutrophil	2547074.75 +/- 836542.25	5738180.89 +/- 2641353.55	3866420.34 +/- 802199.04	3128995.31 +/- 1735260.66	2214850.99 +/- 840392.58	5259655.24 +/- 1361479.42	2944666.50 +/- 1005366.72	2269832.09 +/- 1084821.33
Eosinophil	195722.60 +/- 119931.06	100279.27 +/- 64292.90	94212.17 +/- 55836.45	147081.40 +/- 66594.42	88841.50 +/- 27403.35	57496.25 +/- 28396.90	48451.18 +/- 21655.40	62653.72 +/- 26124.36
B cells	115535.59 +/- 57976.33	77568.19 +/- 41584.63	89312.18 +/- 42852.84	118747.59 +/- 70005.19	96165.82 +/- 35613.06	77709.90 +/- 69723.13	76061.29 +/- 45727.35	95072.84 +/- 53696.03

Therapeutic

Cell Type	0hr Untreated	4hr Untreated	24hr Untreated	48hr Untreated	0hr Treated	4hr Treated	24hr Treated	48hr Treated
Neutrophil	2003764.00 +/- 1195907.70	6646634.71 +/- 1793999.42	3858131.55 +/- 1043502.81	2650358.28 +/- 1253299.05	2279707.91 +/- 924942.65	8137764.90 +/- 3071801.72	4380888.53 +/- 1861149.24	2619628.43 +/- 1116279.46
Eosinophil	65604.46 +/- 77356.56	59161.46 +/- 80259.56	82048.13 +/- 117597.84	107093.45 +/- 144594.90	89266.36 +/- 80901.99	76746.01 +/- 53832.06	80196.66 +/- 75411.85	129348.80 +/- 142997.66
CD4 T cell	476038.31 +/- 180692.74	290370.53 +/- 86552.49	285085.76 +/- 97977.00	414782.76 +/- 238595.92	521125.26 +/- 348115.54	358384.85 +/- 182923.81	407767.11 +/- 157857.74	365355.63 +/- 98722.24
CD8 T cell	240006.56 +/- 147139.03	136819.56 +/- 71972.94	163149.86 +/- 75851.58	213153.23 +/- 155009.88	275976.86 +/- 109223.61	195028.18 +/- 74738.32	242629.40 +/- 67854.79	233306.20 +/- 96027.19
Natural Killer cell	132858.95 +/- 67786.86	121055.06 +/- 106192.30	143642.44 +/- 93844.28	149178.32 +/- 94191.90	88427.61 +/- 65205.10	77126.10 +/- 45311.90	90308.68 +/- 50585.20	109124.95 +/- 100744.03
Plasmacytoid Dendritic Cell	9314.52 +/- 5928.65	9247.36 +/- 4896.58	11529.55 +/- 4899.29	11324.81 +/- 5446.37	4856.87 +/- 1573.46	6782.25 +/- 2560.22	6283.16 +/- 2848.51	7508.95 +/- 5517.02
Classical Dendritic Cell 1	1056.78 +/- 623.26	1000.09 +/- 632.66	1510.58 +/- 1005.48	1632.17 +/- 1321.34	722.02 +/- 387.20	508.25 +/- 216.11	553.92 +/- 495.30	1101.13 +/- 956.49
Classical Dendritic Cell 2	5112.44 +/- 2681.07	6573.38 +/- 2870.89	5654.89 +/- 3834.42	11447.18 +/- 11652.67	4064.48 +/- 1991.11	4814.88 +/- 1941.82	3178.69 +/- 1428.56	3516.10 +/- 1863.77
B cells	133645.00 +/- 71925.39	94552.58 +/- 49289.85	73979.17 +/- 27958.70	124746.12 +/- 67536.89	115666.52 +/- 43817.93	100827.27 +/- 42160.17	108533.73 +/- 28391.22	118430.57 +/- 29700.16
FOXP3 T cells	1125.30 +/- 1170.50	798.18 +/- 1257.25	847.48 +/- 792.27	852.87 +/- 771.51	113.41 +/- 133.98	65.15 +/- 44.07	250.94 +/- 402.36	1008.33 +/- 1854.95

Supplementary Figure 5 – Changes to leukocyte numbers in peripheral blood with prophylactic and therapeutic dosing with GSK2256294 during inflammation.
The forearms of participants were intradermally injected with UV-killed *E. coli* (UVKEc) resulting in a local and peripheral inflammatory response. 2 hrs prior to (prophylactic) or 4 hrs after (therapeutic) UVKEc injection, participants were dosed with 15 mg of GSK2256294, a soluble epoxide hydrolase inhibitor. Peripheral blood was collected, and leukocytes analysed at baseline and 4 hrs, 24 hrs and 48 hrs post UVKEc injection using multiparameter flow cytometry. Data are presented as mean cell number per ml ± SD.

Supplementary, Figure 6

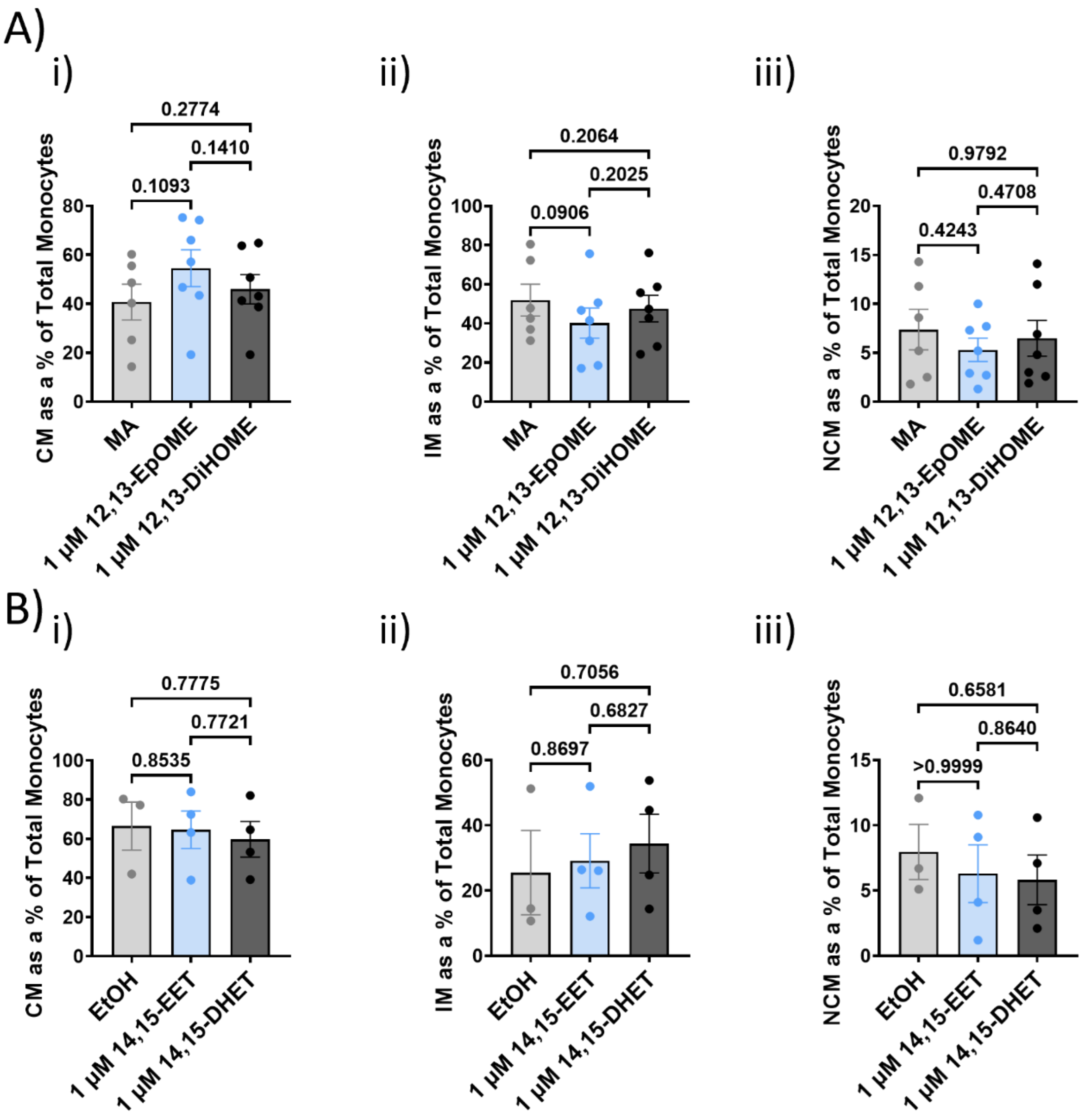

Supplementary Figure 6 – Effect of 12,13-EpOME and 12,13-DiHOME on monocyte differentiation *in vitro*.

PBMCs (2×10^6/well) were seeded and treated with 1 µM epoxy-oxylipin or vehicle for 24 h. Cells were phenotyped by flow cytometry. (A) (i) % classical monocytes (CM), (ii) % intermediate monocytes (IM), (iii) % non-classical monocytes (NCM) with 1 µM 12,13-EpOME, 1 µM 12,13-DiHOME or MA (MA: n = 6; 12,13-EpOME and 12,13-DiHOME: n = 7; biologically independent samples). (B) (i) % CM, (ii) IM, and (iii) NCM with 1 µM 14,15-EET, 1 µM 14,15-DHET or EtOH (EtOH: n = 3; 14,15-EET and 14,15-DHET: n = 4; biologically independent samples). Data were assessed for normalisation using the D'Agostino & Pearson test, Shapiro-Wilk test and visualised using a QQ plot. Parametric data are presented as mean ± SD. Data were analysed using a one-way-ANOVA. Source data are provided as a Source Data file.

Supplementary, Figure 7

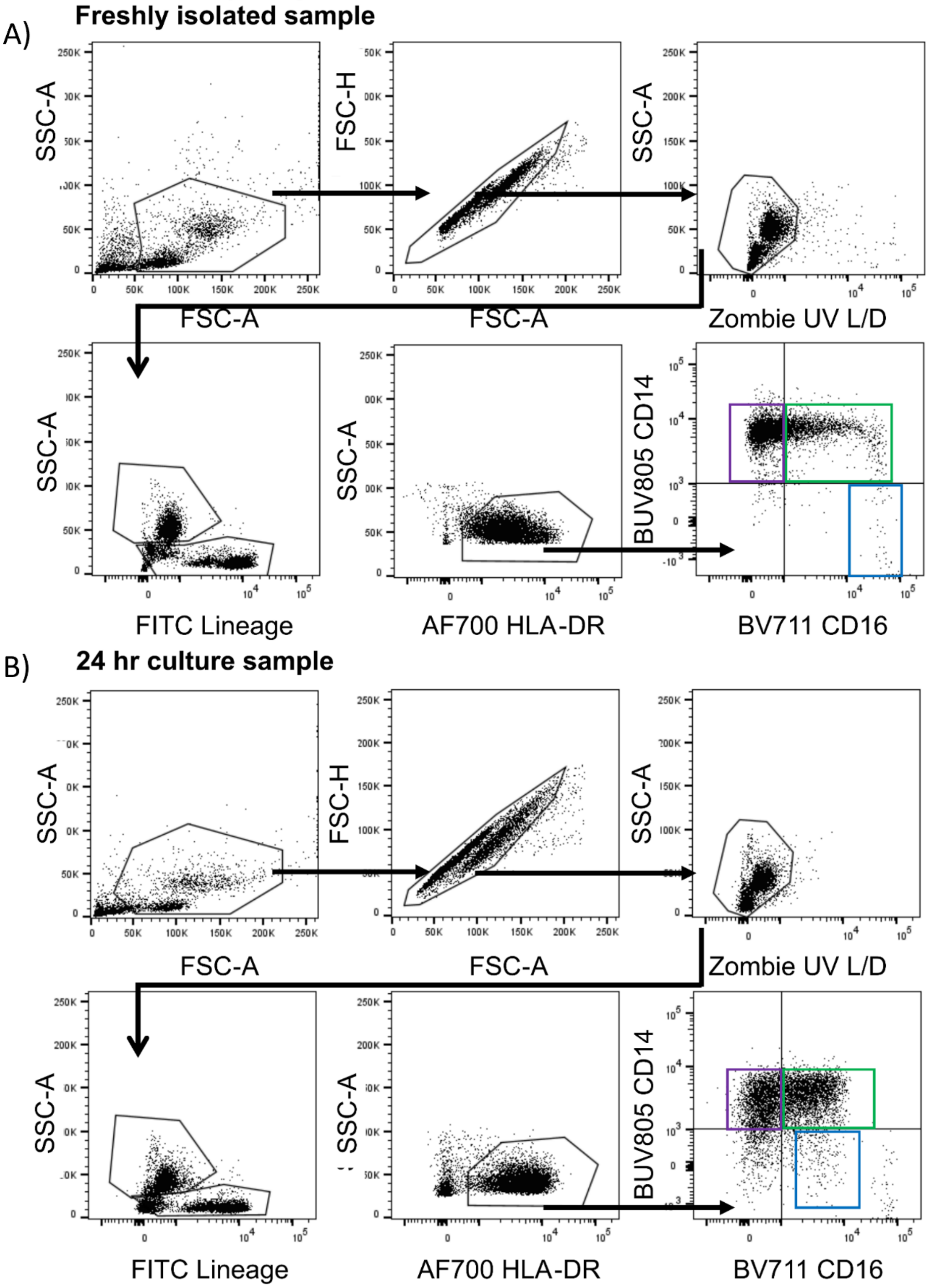

Supplementary Figure 7 - Flow cytometry gating strategies for identification of monocyte subsets in (A) freshly isolated peripheral blood and (B) after 24 hrs in culture.

An initial gate around all leukocytes assigned. These cells are taken forward to exclude doublets and dead cells using Zombie UV. NK cells, T cells and B cells are removed from the sample by gating for the FITC positive cells, which includes markers for CD3, CD56 and CD19. The FITC negative cells are taken forward and gated for AF700. Classical (CD14+CD16-), Intermediate (CD14+CD16+) and Non-Classical (CD14-CD16+) subsets are assigned according to CD14 and CD16 expression.

Supplementary, Figure 8

A)

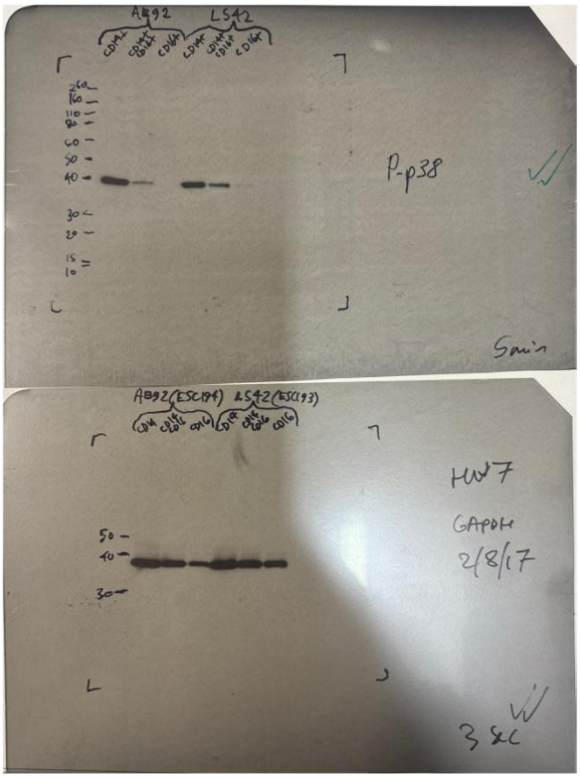

B)

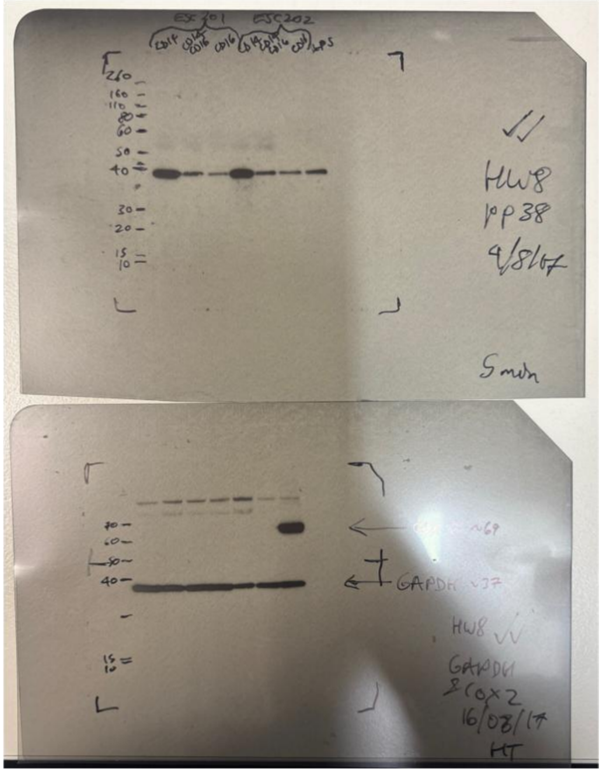

Supplementary Figure 8 – Western blots images for P-p38 and GAPDH

(A) Expression of P-p38 and GAPDH in freshly isolated monocyte subsets for donors AB92 and L542.
(B) Expression of P-p38 and GAPDH in freshly isolated monocyte subsets for donors ESC201 and ESC202.

Supplementary, Figure 9

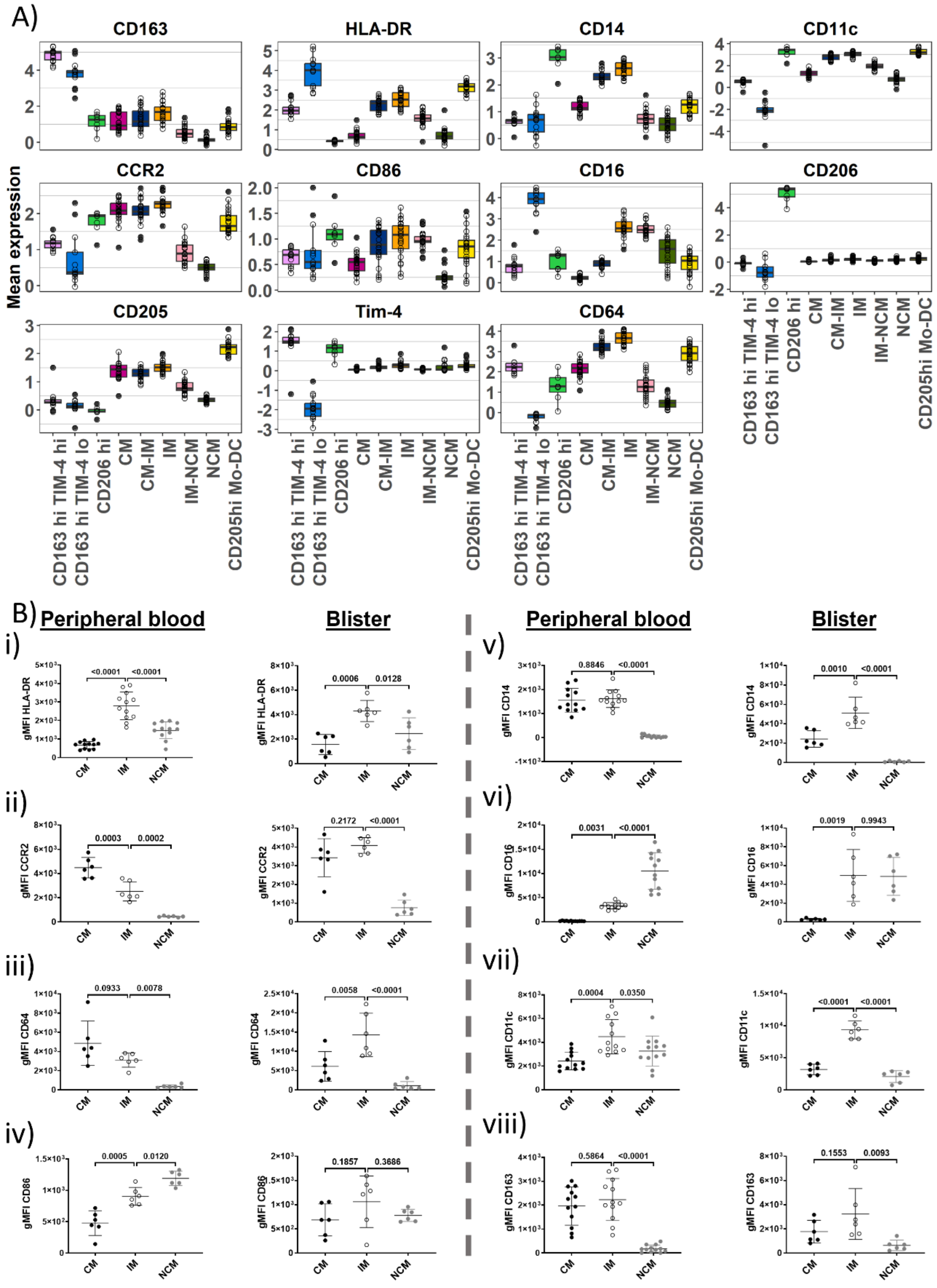

Supplementary Figure 9 – Marker expression in mononuclear phagocyte clusters in the blister

(A) Boxplots of marker expression in mononuclear phagocyte populations in the blister. Total monocytes were extracted from FCS files in FlowJo and then subsequently clustered using the package CATALYST based on the expression of CD163, HLA-DR, CD14, CD16, CD11c, CCR2, CD86, CD16, CD206, CD205, Tim-4 and CD64. Eight monocyte populations were identified and labelled (n = 6; biologically independent samples). (B) Monocyte phenotype in blood vs. blister 24 hrs after inflammatory onset. The forearms of participants were intradermally injected with UV-killed *E. coli* (UVKEc) resulting in a local and peripheral inflammatory response. Peripheral bloods and local inflammatory exudate were collected and analysed at baseline and 4 hrs, 24 hrs and 48 hrs post UVKEc injection. Data shows monocyte expression for (i) HLA-DR, (ii) CCR2, (iii) CD64, (iv) CD86, (v) CD14, (vi) CD16, (vii) CD11c and (viii) CD163 on peripheral blood or blister monocyte subsets at 24 hrs post UVKEc. Data are presented as mean ± SD. Peripheral blood HLA-DR, CD14, CD16, CD11c and CD163 n = 12 (biologically independent samples). Peripheral blood CCR2, CD64, CD86 n = 6 (biologically independent samples). Blister samples n = 6 (biologically independent samples). Data were analysed using a one-way-ANOVA. Source data are provided as a Source Data file.

Supplementary, Figure 10

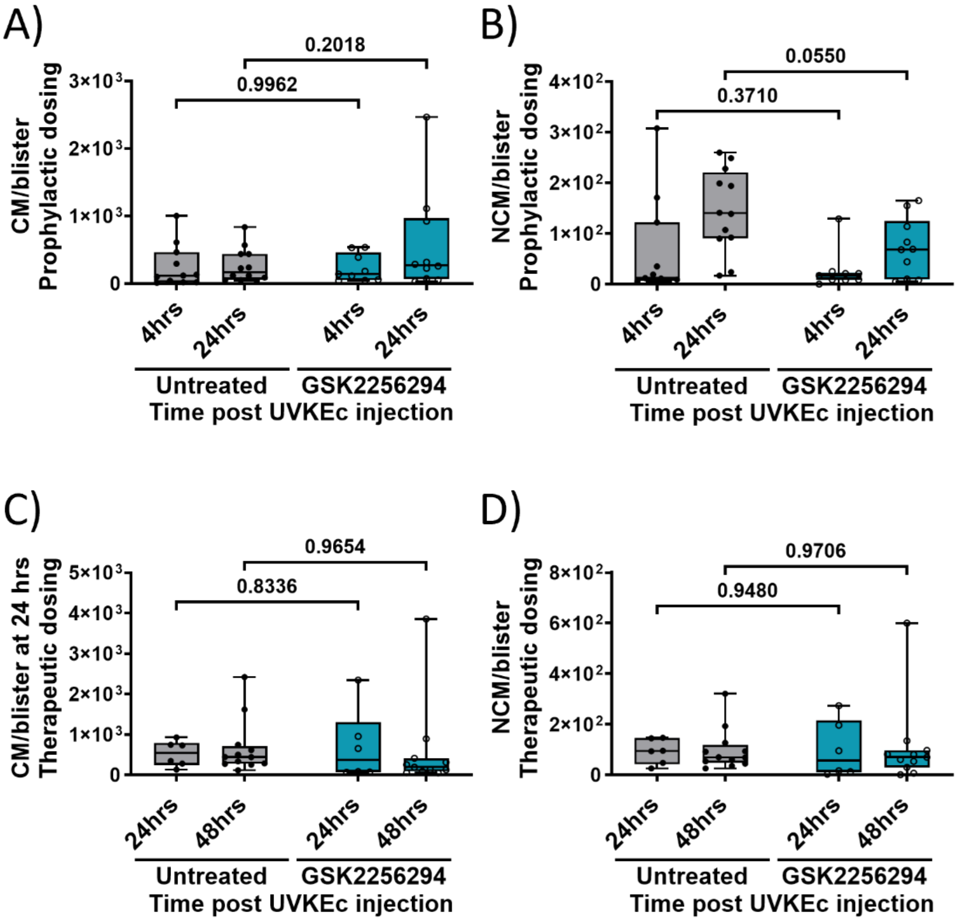

Supplementary Figure 10 – Classical and Non-classical monocytes per blister in the prophylactic and therapeutic arms of the study.

The forearms of participants were intradermally injected with UV-killed *E. coli* (UVKEc) resulting in a local inflammatory response. 2 hrs prior to (prophylactic) or 4 hrs after (therapeutic) UVKEc injection, participants were dosed with 15 mg of GSK2256294. Local inflammatory exudate was collected and subject to lipidomic and flow cytometric analysis at 4 and 24 hrs (prophylactic) and 24 and 48 hrs (therapeutic).

Classical monocytes (CD14+CD16-), intermediate monocytes (CD14+CD16+) and non-classical monocytes (CD14-CD16+) were quantified using manual gating in FlowJo. (A) Classical monocytes/blister at 4 and 24 hrs (prophylactic) (untreated: n = 12; GSK2256294: n = 10; biologically independent samples). (B) Non-classical monocytes/blister at 4 and 24 hrs (prophylactic) (untreated: n = 12; GSK2256294: n = 10; biologically independent samples). (C) Classical monocytes/blister at 24 and 48 hrs (therapeutic) (untreated: n = 6-12; GSK2256294: n = 6-11; biologically independent samples). (D) Non-classical monocytes/blister at 24 and 48 hrs (therapeutic) (untreated: n = 6-12; GSK2256294: n = 6-11; biologically independent samples). Normality was assessed using the D'Agostino & Pearson test, Shapiro-Wilk test and visualised using a QQ plot. Box-and-whisker plots show the median (center line), the interquartile range (25th–75th percentiles; box), and the full data range (whiskers, minimum to maximum). Data were analysed using two-way-ANOVA mixed effect analysis with Uncorrected Fisher's LSD. Source data are provided as a Source Data file.

Supplementary, Figure 11

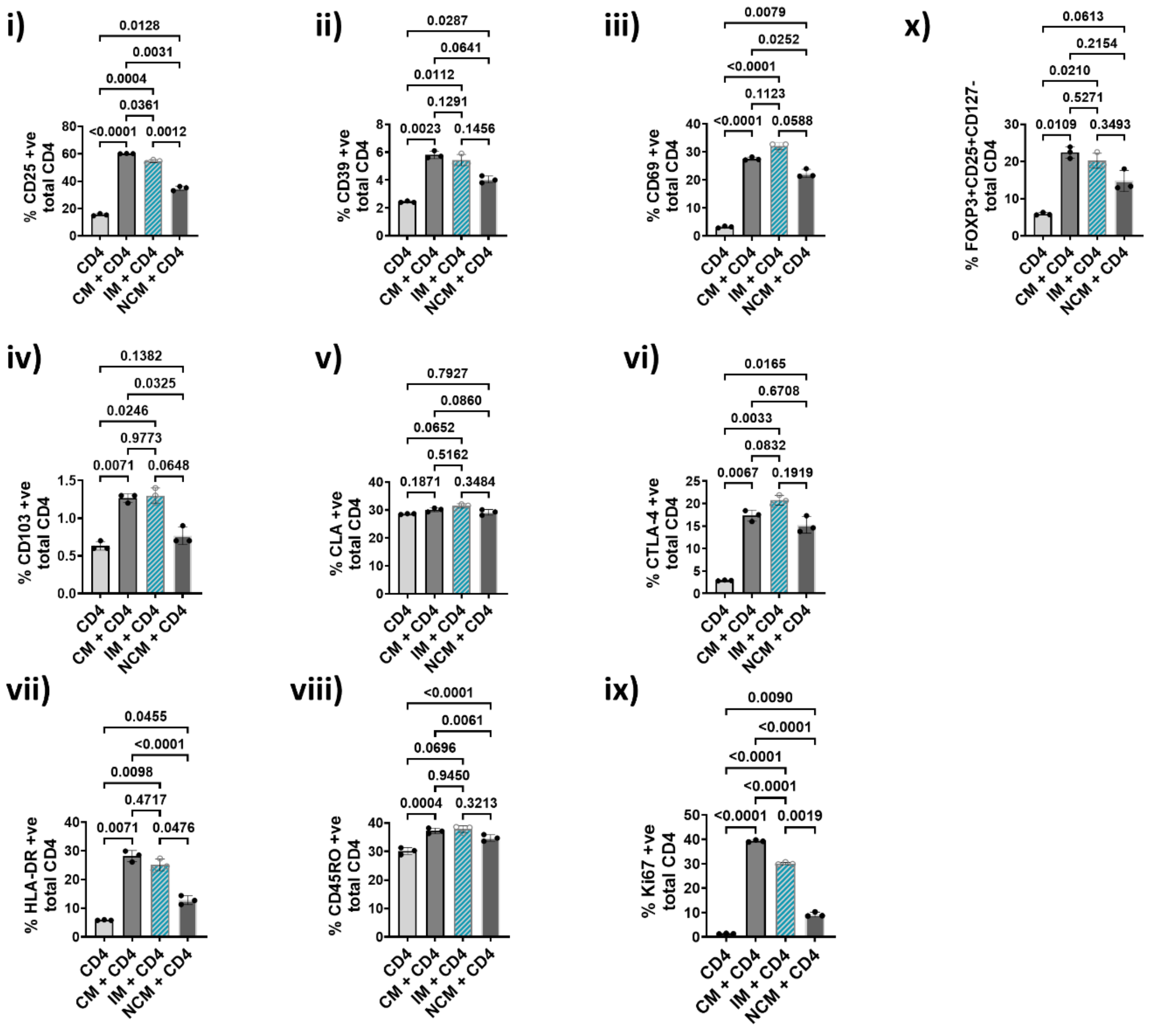

Supplementary Figure 11 – CD4 T cell phenotype after co-culture with monocyte subsets

Classical, intermediate and non-classical monocytes were sorted using FACS and co-cultured with CD4 T cells at a 5:1 (T cell:monocyte) ratio for 48 hours and subsequently analysed by multi-parameter spectral flow cytometry. The % expression on total CD4 cells was visualised for (i) CD25, (ii) CD39, (iii) CD69, (iv) CD103, (v) CLA, (vi) CTLA-4, (vii) HLA-DR, (viii) CD45RO, (ix) Ki67 and (x) FOXP3+CD25+CD127- (n = 3; technical repeat). Parametric data are presented as mean ± SD. Data were analysed using a one-way-ANOVA. Source data are provided as a Source Data file.

Supplementary, Figure 12

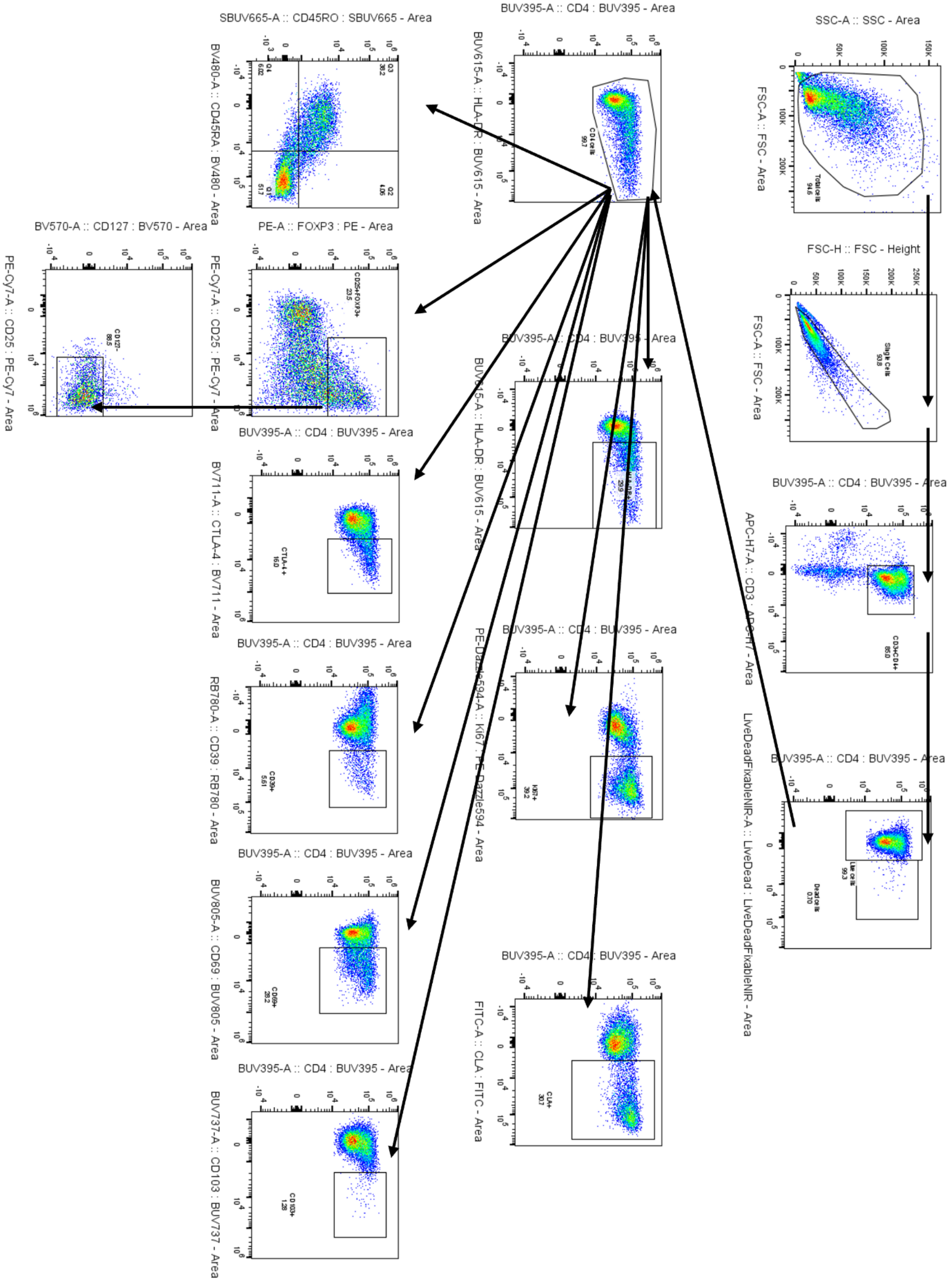

Supplementary Figure 12 – Gating strategy for CD4 phenotyping flow cytometry experiments

Plot representative of a PBMC sample. An initial gate around all leukocytes assigned. These cells are taken forward to exclude doublets. CD4+ T cells were gating based on the expression of CD4 and CD3. Dead cells were excluded using Zombie NIR. Representative plots of expression on total CD4 cells are shown for HLA-DR, Ki67, CLA, CD45RO, CTLA-4, CD39, CD69, CD103. CD25+FOXP3+ cells were gated and these cells were taken forward and a gate on the CD127 cells was assigned. These cells were designated CD25+FOXP3+CD127- T-regulatory cells.

Supplementary, Figure 13

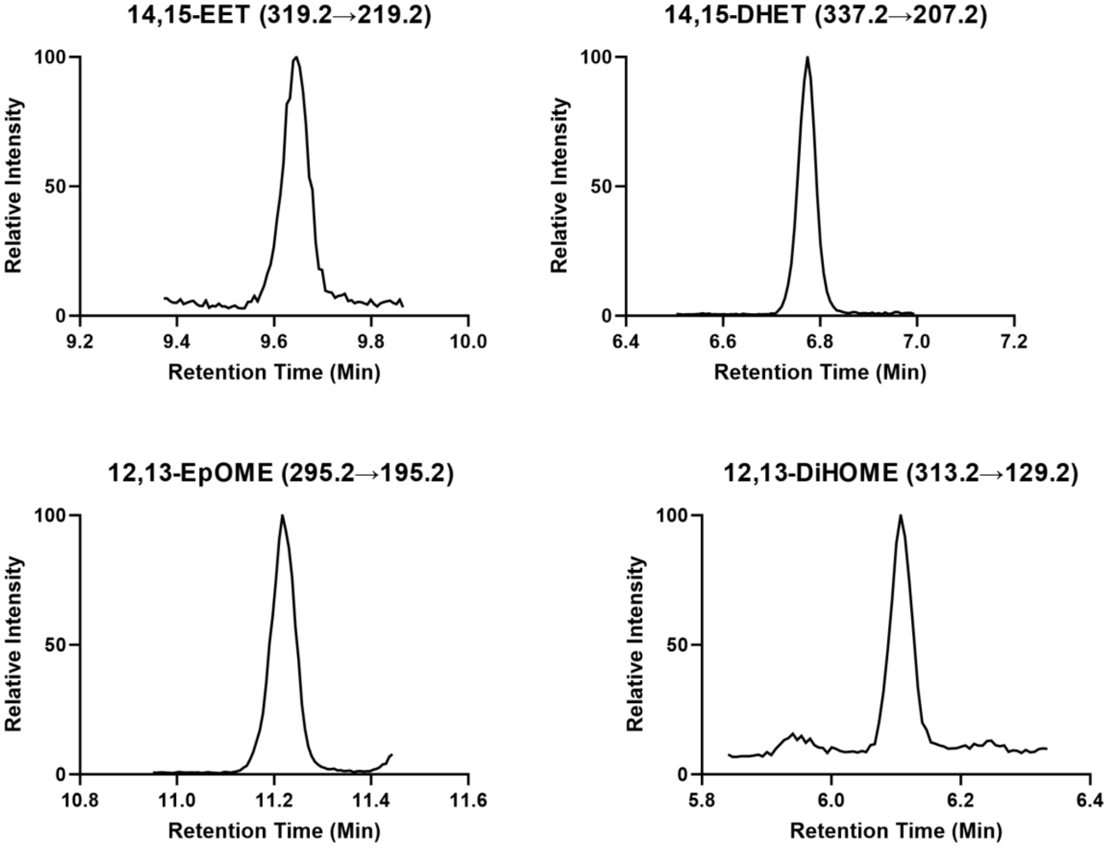

Supplementary Figure 13 -Chromatograms of select plasma LCMS analytes. LCMS analysis of plasma oxylipins was performed as described in methods. Representative chromatograms of an extracted plasma sample for 14,15-EET (m/z transition 319.2→219.2), 14,15-DHET (m/z transition 337.2→207.2), 12,13-EpOME (m/z transition 295.2→195.2) and 12,13-DiHOME (m/z transition 313.2→129.2) are shown as relative intensity (max value for each peak set to 100).

Supplementary, Figure 14

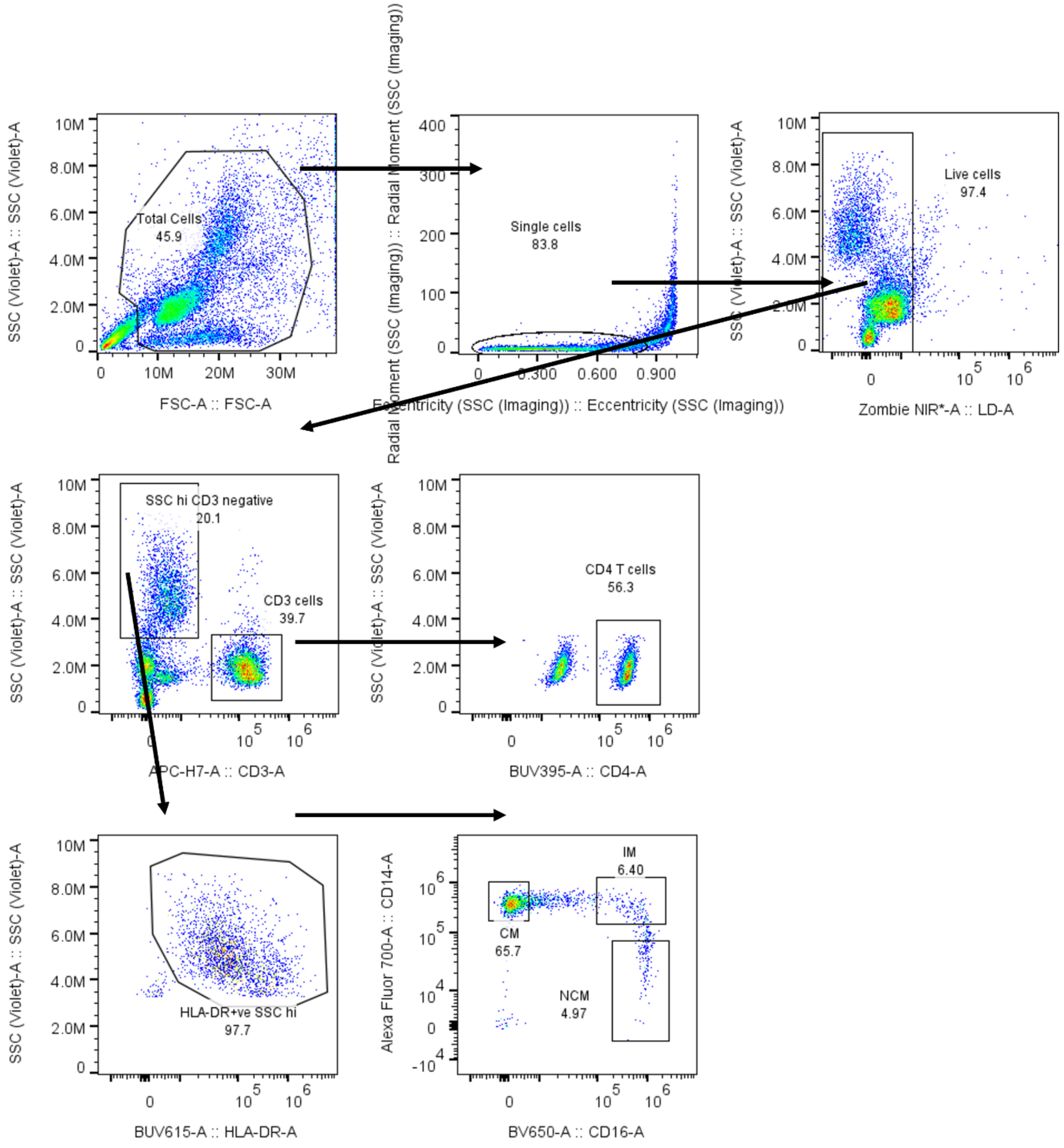

Supplementary Figure 14 - Gating strategy to FACS sort monocytes and CD4 T cells from PBMCs.

Plot representative of a PBMC sample. An initial gate around all leukocytes assigned. These cells are taken forward to exclude doublets and dead cells using Zombie NIR. CD3+CD4+ positive cells are gated for using CD3 on APC-H7 and CD4 on BUV395. Monocytes are gated as CD3-ve SSC hi HLA-DR+ve and then into the three monocyte subsets based on the expression of CD14 on AF700 and CD16 on BV650. Classical monocytes (CM) are defined as CD14+CD16-, intermediate monocytes are defined as CD14+CD16+ and non-classical monocytes (NCM) are defined as CD14-CD16+.
