# Supplementary material for: Epoxy-oxylipins direct monocyte fate in inflammatory resolution in humans
Source: Nat Commun. 2026 Jan 16;17:431. doi: 10.1038/s41467-025-67961-5 (PMC12811254; doi:10.1038/s41467-025-67961-5)
Supplement: Supplementary file 3 — Reporting Summary [file 41467_2025_67961_MOESM3_ESM.pdf]

nature portfolio

Corresponding author(s): Derek W Gilroy

Last updated by author(s): Oct 7, 2025

Reporting Summary

Nature Portfolio wishes to improve the reproducibility of the work that we publish. This form provides structure for consistency and transparency in reporting. For further information on Nature Portfolio policies, see our Editorial Policies and the Editorial Policy Checklist.

Statistics

For all statistical analyses, confirm that the following items are present in the figure legend, table legend, main text, or Methods section.

n/a	Confirmed	
☐	☒	The exact sample size (*n*) for each experimental group/condition, given as a discrete number and unit of measurement
☐	☒	A statement on whether measurements were taken from distinct samples or whether the same sample was measured repeatedly
☐	☒	The statistical test(s) used AND whether they are one- or two-sided<br>*Only common tests should be described solely by name; describe more complex techniques in the Methods section.*
☐	☒	A description of all covariates tested
☐	☒	A description of any assumptions or corrections, such as tests of normality and adjustment for multiple comparisons
☐	☒	A full description of the statistical parameters including central tendency (e.g. means) or other basic estimates (e.g. regression coefficient) AND variation (e.g. standard deviation) or associated estimates of uncertainty (e.g. confidence intervals)
☐	☒	For null hypothesis testing, the test statistic (e.g. *F*, *t*, *r*) with confidence intervals, effect sizes, degrees of freedom and *P* value noted<br>*Give P values as exact values whenever suitable.*
☒	☐	For Bayesian analysis, information on the choice of priors and Markov chain Monte Carlo settings
☒	☐	For hierarchical and complex designs, identification of the appropriate level for tests and full reporting of outcomes
☒	☐	Estimates of effect sizes (e.g. Cohen's *d*, Pearson's *r*), indicating how they were calculated

Our web collection on statistics for biologists contains articles on many of the points above.

Software and code

Policy information about availability of computer code

Data collection	FACS Diva software and ID7000 Sony Software was used for the collection of flow cytometry data and cell sorting populations.
Data analysis	Data was analysed using Graphpad Prism 10.4.7. R studio 2025.05.0 was also used for all R data analysis. CATALYST version 1.26.1 was used within R studio for analysis of flow cytometry data. FlowJo v10.10 was used for analysis of FCS files. NDP view v2.2 and ImageJ v2 were used to analyse IHC images. Other R package files used include ggplot2 (3.5.1), pheatmap (1.0.12), dplyr (1.1.4).

For manuscripts utilizing custom algorithms or software that are central to the research but not yet described in published literature, software must be made available to editors and reviewers. We strongly encourage code deposition in a community repository (e.g. GitHub). See the Nature Portfolio guidelines for submitting code & software for further information.

Data

Policy information about availability of data

All manuscripts must include a data availability statement. This statement should provide the following information, where applicable:

- Accession codes, unique identifiers, or web links for publicly available datasets
- A description of any restrictions on data availability
- For clinical datasets or third party data, please ensure that the statement adheres to our policy

Data supporting the findings of this study are available within the paper and its supplementary information. The raw LCMS files, protocols and metadata were uploaded to MassIVE (https://massive.ucsd.edu/). The accession numbers are MSV000099360 and MSV000099361. Raw FCS files and IHC files are available upon request. Source data are provided with this paper.

Research involving human participants, their data, or biological material

Policy information about studies with human participants or human data. See also policy information about sex, gender (identity/presentation), and sexual orientation and race, ethnicity and racism.

Reporting on sex and gender	In line with our ethical approval, we recruited healthy, male volunteers into the study. Overall we recruited 49 males into this study. Their sex was self reported. These participants were consented individually to take part in this study. Females were not included in this study design due to the use of GSK2256294, which had been through Phase II clinical trialling, but was not licenced.
Reporting on race, ethnicity, or other socially relevant groupings	We recruited participants from all backgrounds, however, as this is not a clinical trial this information has not been included in the manuscript. Although, we note that the majority of participants were from Caucasian backgrounds.
Population characteristics	The participants recruited into this study were all of the male sex (XY background) with a mean age of 24 years.
Recruitment	Participants were recruited using paper flyers that were signposted around the UCL campus. This led to a cohort of males being recruited that were between the ages of 18-30 due to recruitment taking place on the university campus.
Ethics oversight	All studies were approved by the UCL Research Ethics Committee (Project ID number: 1309/004). All procedures performed were in line with the ethical standards of the UCL Institutional Committee and according to the principles of the declaration of Helsinki.

Note that full information on the approval of the study protocol must also be provided in the manuscript.

Field-specific reporting

Please select the one below that is the best fit for your research. If you are not sure, read the appropriate sections before making your selection.

[x] Life sciences [ ] Behavioural & social sciences [ ] Ecological, evolutionary & environmental sciences

For a reference copy of the document with all sections, see nature.com/documents/nr-reporting-summary-flat.pdf

Life sciences study design

All studies must disclose on these points even when the disclosure is negative.

Sample size	The sample size was determined to be 12 participants in each arm of the study. I.e. 12 participants in the untreated arm of the prophylactic study, 12 in the treated arm and similarly for the therapeutic arm of the study. During the study, there were instances when participants refused blood withdrawal and/or there were technical difficulties in obtaining cells from the tissue. Therefore, the data points are varied in the analysis of this work.
Data exclusions	No data was excluded from this study.
Replication	All experiments were repeated using experimental repeats to verify the reproducibility of the results in this study.
Randomization	There was no randomisation into the individual groups in this study.
Blinding	There was no blinding in the administration of the drug in this study as there was no placebo control available. All lipidomic analysis was blinded.

Reporting for specific materials, systems and methods

We require information from authors about some types of materials, experimental systems and methods used in many studies. Here, indicate whether each material, system or method listed is relevant to your study. If you are not sure if a list item applies to your research, read the appropriate section before selecting a response.

Materials & experimental systems

n/a	Involved in the study	
[ ]	[x]	Antibodies
[ ]	[x]	Eukaryotic cell lines
[x]	[ ]	Palaeontology and archaeology
[x]	[ ]	Animals and other organisms
[x]	[ ]	Clinical data
[x]	[ ]	Dual use research of concern
[x]	[ ]	Plants

Methods

n/a	Involved in the study	
[x]	[ ]	ChIP-seq
[ ]	[x]	Flow cytometry
[x]	[ ]	MRI-based neuroimaging

Antibodies

Antibodies used	FITC CD3 300306, FITC CD19 302206, FITC CD20 302304, FITC CD56 392414, FITC CD66b 305104, PerCP-Cy5.5 CD56 318322, PerCP-Cy5.5 CD141 344112, PerCP-Cy5.5 CD11b 301328, BV421 CD11c 301628, BV421 CD62L 304828, BV510 CCR2 357218, BV510 CD11b 301628, BV510 HLA-DR 307646, BV605 CD86 374214, BV605 CD25 302632, BV711 CD16 302044, BV785 CD163 333632, BV785 CD206 321142, BV786 CD27 563327, BUV395 CD123 564195, BUV395 CD4 564724, BUV395 CD1a 756536, APC CD163 333610, APC CD207 352205, AF700 HLA-DR 307626, AF700 CD66b 305114, APC-Cy7 CD1c 331520, APC-Cy7 CD8 301016, APC-Cy7 CD206 321120, PE CD205 359204, PE FOXP3 320107, PE MerTK 367608, PE CCR7 535204, PE CD25 302605, PE-Dazzle CD19 302252, PE-Dazzle CD20 302348, PE-Dazzle CD3 300336, PE-Dazzle CD56 392410, PE-Dazzle CD66b 305122, PE-Dazzle CD64 305032, PE-Cy7 TIM-4 354006, PE-Cy7 CD62L 304822, PE-Cy7 CD45RA 304125, BUV805 CD14 612902, BUV805 CD69 748763, Zombie UV Live/Dead 423107, AF647 P-p38 526066, Starbright UV 665 CD45RO MCA461SBUV665, BUV615 HLA-DR 751142, BUV737 CD103 748502, BV421 CCR6 562515, BV480 CD45RA 566155, BV650 CD16 563692, BV711 CTLA-4 369632, FITC CLA 321306, PerCPFluor710 TIGIT 46-9500-42, RB780 CD39 570123, AF700 CD14 325614, APC-H7 CD3 560275, PE FOXP3 12-4777-43, PE-Dazzle Ki67 350533, Zombie NIR Live/Dead 423106.
Validation	All antibodies used in this research are commercially available. Antibodies were validated (both positive and negative controls) and titrated upon receipt based on the manufacturers recommendations.

Eukaryotic cell lines

Policy information about cell lines and Sex and Gender in Research

Cell line source(s)	K562 cell line - derived from the plural effusion of a 53-year old female patient with CML.
Authentication	The cell line was purchased from ATCC, category code CCL-243.
Mycoplasma contamination	The cell line was negative for mycoplasma.
Commonly misidentified lines (See ICLAC register)	*Name any commonly misidentified cell lines used in the study and provide a rationale for their use.*

Plants

Seed stocks	*Report on the source of all seed stocks or other plant material used. If applicable, state the seed stock centre and catalogue number. If plant specimens were collected from the field, describe the collection location, date and sampling procedures.*
Novel plant genotypes	*Describe the methods by which all novel plant genotypes were produced. This includes those generated by transgenic approaches, gene editing, chemical/radiation-based mutagenesis and hybridization. For transgenic lines, describe the transformation method, the number of independent lines analyzed and the generation upon which experiments were performed. For gene-edited lines, describe the editor used, the endogenous sequence targeted for editing, the targeting guide RNA sequence (if applicable) and how the editor was applied.*
Authentication	*Describe any authentication procedures for each seed stock used or novel genotype generated. Describe any experiments used to assess the effect of a mutation and, where applicable, how potential secondary effects (e.g. second site T-DNA insertions, mosiacism, off-target gene editing) were examined.*

Flow Cytometry

Plots

Confirm that:

- [x] The axis labels state the marker and fluorochrome used (e.g. CD4-FITC).
- [x] The axis scales are clearly visible. Include numbers along axes only for bottom left plot of group (a 'group' is an analysis of identical markers).
- [x] All plots are contour plots with outliers or pseudocolor plots.
- [x] A numerical value for number of cells or percentage (with statistics) is provided.

Methodology

Sample preparation

To isolate leukocytes for flow cytometry analysis, 1 ml of whole blood, collected in a EDTA vacutainer tube (4 ml; Griener Bio-One), was added to 9 ml of ammonium-chloride-potassium lysis buffer (ACK; Lonza). Blister cells were obtained via centrifugation of blister exudate. PBMCs were isolated as follows: Peripheral blood was collected in EDTA. Peripheral blood mononuclear cells were isolated by mixing 1:1 with Hanks Buffer Salt Solution (HBSS) (Gibco, 14170). The 1:1 peripheral blood/HBSS solution was layered onto Ficoll-Paque PLUS (17-1440-03) in a 2:1 ratio. Samples were spun at 1000 g for 30 mins with the brakes off. The layer of PBMCs was collected and washed in HBSS followed by a spin at 400 g for 10 mins. Red blood cell contamination was lysed using 1 ml Ack lysis buffer for 5 minutes at RT. Samples were topped up to 30 ml with PBS, cells were enumerated and then spun for a further 5 minutes at 400 g. All samples were stained in 100µl for 30 minutes at 4oC. Antibody cocktails were prepared with 50 µl of Brilliant Stain Buffer (BSB) (BD Biosciences, 566349), antibodies (Supplementary Table 4), with the remainder of the volume made up with FACS buffer (PBS, 5% Foetal Calf Serum, 2 mM EDTA). All panels were compensated appropriately, either using cells as vehicles for single stains, UltraComp eBeadsTM compensation beads (Thermo Fisher, 01-2222-41), or ArCTM Amine Reactive compensation bead kit (Thermo Fisher, A10346). Unstained sample and Fluorescence minus-one (FMO) and, where appropriate, isotype control antibodies were used as a control. For samples with intracellular staining: after the final wash step of the extracellular stain, cells were re-suspended in 200 µl Fix/Perm buffer (eBioscience FOXP3 Fix/Perm Buffer) for 45 minutes, in the dark, at RT. Afterward cells were spun at 1000 g, for 5 minutes, at ET. The supernatant was aspirated, and cells were washed in 200 µl of perm wash buffer (eBioscience FOXP3 Fix/Perm Buffer) and spun at 1000 g, for 5 minutes, at RT. Cells were stained for intracellular antibodies in perm buffer for 45 minutes at RT. Cells were twice washed in 200 µl perm wash buffer and centrifuged at 1000 g for 5 minutes. Cells were resuspended in 150 µl FACS buffer and 150 µl perm wash buffer for data collection on the flow cytometer.

Instrument

Data were acquired using a BD Fortessa UV X20 flow cytometer or a Sony ID7000 spectral flow cytometer. Cell sorting was performed on a BD S8 Spectral cell sorter.

Software

Analysis of flow cytometry data used either FlowJo V10.10 or ID7000 analysis software. Further unbiased analysis was performed using the R package CATALYST. Data acquisition software was either BD FACS DIVA, ID7000 Acquisition software or FACS Discover.

Cell population abundance

Blister cell numbers were quantified by acquiring the entire sample on the flow cytometer. Peripheral blood samples were quantified either by manual haemocytometer or CountBright cell counting beads (Invitrogen). Cell abundance in post-sort populations was determined using the gating strategy shown in supplementary figure 17 and cell populations were counted used a manual haemocytometer post sort to confirm cell numbers. CD4 cells were on average 50% of total CD3 cells. Classical monocytes were approximately 65% of total monocytes, Intermediate monocytes were approximately 7% of total monocytes and non-classical monocytes were approx 5% of total monocytes. The gates used to cell sort the populations were stringent.

Gating strategy

All gating strategies are described in the supplementary information.

Flow cytometry gating strategies for identification of monocyte subsets in peripheral blood and blister:

Identification of monocyte subsets from peripheral blood for analysis in FlowJo. An initial gate around all leukocytes assigned. These cells are taken forward to exclude doublets and dead cells. NK cells, T cells, B cells and neutrophils are removed from the sample by gating for the FITC positive cells, which includes markers for CD3, CD56, CD19, CD20 and CD66b. T cells and B cells are gated in orange and NK cells in yellow. Neutrophils are assigned based on high SSC and high CD16 expression. The FITC negative cells are taken forward and gated for AF700 HLA-DR positive cells. Classical (CD14+CD16-), Intermediate (CD14 +CD16+) and Non-Classical (CD14-CD16+) subsets are assigned according to CD14 and CD16 expression. pDCs are taken from the CD14-CD16- and assigned as CD123+. cDCs are taken from the CD123- cells and assigned according to expression of CD141 (cDC1) and CD1c (cDC2).

Identification of HLA-DR+CD14+CD16+ monocytes from peripheral blood for input into CATALYST for supervised clustering. An initial gate around all leukocytes assigned. These cells are taken forward to exclude doublets and dead cells. NK cells, T cells, B cells and neutrophils are removed from the sample by gating for the FITC positive cells, which includes markers for CD3, CD56, CD19, CD20 and CD66b. T cells and B cells are gated in orange and NK cells in yellow. Neutrophils are assigned based on high SSC and high CD16 expression. The FITC negative cells are taken forward and gated for AF700 HLA-DR positive cells. Monocytes that are used in the supervised clustering are gated in green.

Identification of monocyte subsets from the blister for analysis in FlowJo. An initial gate around all leukocytes assigned. These cells are taken forward to exclude doublets and dead cells. NK cells, T cells, B cells and neutrophils are removed from the sample by gating for the FITC positive cells, which includes markers for CD3, CD56, CD19, CD20 and CD66b. T cells and B cells are gated in orange and NK cells in yellow. Neutrophils are assigned based on high SSC and high CD16 expression. The FITC negative cells are taken forward and gated for AF700 HLA-DR positive cells. Classical (CD14+CD16-), Intermediate (CD14 +CD16+) and Non-Classical (CD14-CD16+) subsets are assigned according to CD14 and CD16 expression. pDCs are taken from the CD14-CD16- and assigned as CD123+. cDCs are taken from the CD123- cells and assigned according to expression of CD141 (cDC1) and CD1c (cDC2).

Identification of HLA-DR+CD14+CD16+ monocytes from the blister for input into CATALYST for supervised clustering. An initial gate around all leukocytes assigned. These cells are taken forward to exclude doublets and dead cells. NK cells, T cells, B cells and neutrophils are removed from the sample by gating for the FITC positive cells, which includes markers for CD3, CD56, CD19, CD20 and CD66b. T cells and B cells are gated in orange and NK cells in yellow. Neutrophils are assigned based on high SSC and high CD16 expression. The FITC negative cells are taken forward and gated for AF700 HLA-DR positive cells. Monocytes that are used in the supervised clustering are gated in green.

Flow cytometry gating strategies for identification of monocyte subsets in (A) freshly isolated peripheral blood and (B) after 24 hrs in culture:

An initial gate around all leukocytes assigned. These cells are taken forward to exclude doublets and dead cells using Zombie UV. NK cells, T cells and B cells are removed from the sample by gating for the FITC positive cells, which includes markers for CD3, CD56 and CD19. The FITC negative cells are taken forward and gated for AF700. Classical (CD14+CD16-), Intermediate (CD14+CD16+) and Non-Classical (CD14-CD16+) subsets are assigned according to CD14 and CD16 expression.

Gating strategy to FACS sort CD4 cells and monocytes from PBMCs:

Plot representative of a PBMC sample. An initial gate around all leukocytes assigned. These cells are taken forward to exclude doublets and dead cells using Zombie NIR. CD3+CD4+ positive cells are gated for using CD3 on APC-H7 and CD4 on BUV395. Monocytes are gated as CD3-ve SSC hi HLA-DR+ve and then into the three monocyte subsets based on the expression of CD14 and CD16. Classical monocytes (CM) are defined as CD14+CD16-, intermediate monocytes are defined as CD14+CD16+ and non-classical monocytes (NCM) are defined as CD14-CD16+.

Gating Strategy for phenotyping of CD4 T cells:

An initial gate around all leukocytes assigned. These cells are taken forward to exclude doublets. CD4+ T cells were gating based on the expression of CD4 and CD3. Dead cells were excluded using Zombie NIR. Representative plots of expression on total CD4 cells are shown for HLA-DR, Ki67, CLA, CD45RO, CTLA-4, CD39, CD69, CD103. CD25+FOXP3+ cells were gated and these cells were taken forward and a gate on the CD127 cells was assigned. These cells were designated CD25+FOXP3 +CD127- T-regulatory cells.

☒ Tick this box to confirm that a figure exemplifying the gating strategy is provided in the Supplementary Information.
